# Sustained pigmentation causes DNA damage and invokes translesion polymerase Polκ for repair in melanocytes

**DOI:** 10.1093/nar/gkad704

**Published:** 2023-09-11

**Authors:** Madeeha Ghazi, Shivangi Khanna, Yogaspoorthi Subramaniam, Jeyashri Rengaraju, Farina Sultan, Iti Gupta, Kanupriya Sharma, Sudhir Chandna, Rajesh S Gokhale, Vivek T Natarajan

**Affiliations:** CSIR-Institute of Genomics and Integrative Biology, Mathura Road, New Delhi 110025, India; Academy of Scientific and Innovative Research (AcSIR), Ghaziabad, Uttar Pradesh 201002, India; CSIR-Institute of Genomics and Integrative Biology, Mathura Road, New Delhi 110025, India; Academy of Scientific and Innovative Research (AcSIR), Ghaziabad, Uttar Pradesh 201002, India; CSIR-Institute of Genomics and Integrative Biology, Mathura Road, New Delhi 110025, India; Academy of Scientific and Innovative Research (AcSIR), Ghaziabad, Uttar Pradesh 201002, India; CSIR-Institute of Genomics and Integrative Biology, Mathura Road, New Delhi 110025, India; Academy of Scientific and Innovative Research (AcSIR), Ghaziabad, Uttar Pradesh 201002, India; CSIR-Institute of Genomics and Integrative Biology, Mathura Road, New Delhi 110025, India; Academy of Scientific and Innovative Research (AcSIR), Ghaziabad, Uttar Pradesh 201002, India; CSIR-Institute of Genomics and Integrative Biology, Mathura Road, New Delhi 110025, India; Academy of Scientific and Innovative Research (AcSIR), Ghaziabad, Uttar Pradesh 201002, India; Institute of Nuclear Medicine and Allied Sciences, Defence Research and Development Organization, Delhi 110054, India; Institute of Nuclear Medicine and Allied Sciences, Defence Research and Development Organization, Delhi 110054, India; CSIR-Institute of Genomics and Integrative Biology, Mathura Road, New Delhi 110025, India; National Institute of Immunology, Aruna Asaf Ali Marg, New Delhi 110067, India; CSIR-Institute of Genomics and Integrative Biology, Mathura Road, New Delhi 110025, India; Academy of Scientific and Innovative Research (AcSIR), Ghaziabad, Uttar Pradesh 201002, India

## Abstract

Melanin protects skin cells from ultraviolet radiation-induced DNA damage. However, intermediates of eumelanin are highly reactive quinones that are potentially genotoxic. In this study, we systematically investigate the effect of sustained elevation of melanogenesis and map the consequent cellular repair response of melanocytes. Pigmentation increases γH2AX foci, DNA abasic sites, causes replication stress and invokes translesion polymerase Polκ in primary human melanocytes, as well as mouse melanoma cells. Confirming the causal link, CRISPR-based genetic ablation of tyrosinase results in depigmented cells with low Polκ levels. During pigmentation, Polκ activates replication stress response and keeps a check on uncontrolled proliferation of cells harboring melanin-damaged DNA. The mutational landscape observed in human melanoma could in part explain the error-prone bypass of DNA lesions by Polκ, whose absence would lead to genome instability. Thereby, translesion polymerase Polκ is a critical response of pigmenting melanocytes to combat melanin-induced DNA alterations. Our study illuminates the dark side of melanin and identifies (eu)melanogenesis as a key missing link between tanning response and mutagenesis, mediated *via* the necessary evil translesion polymerase, Polκ.

## Graphical Abstract

**Graphical Abstract:**
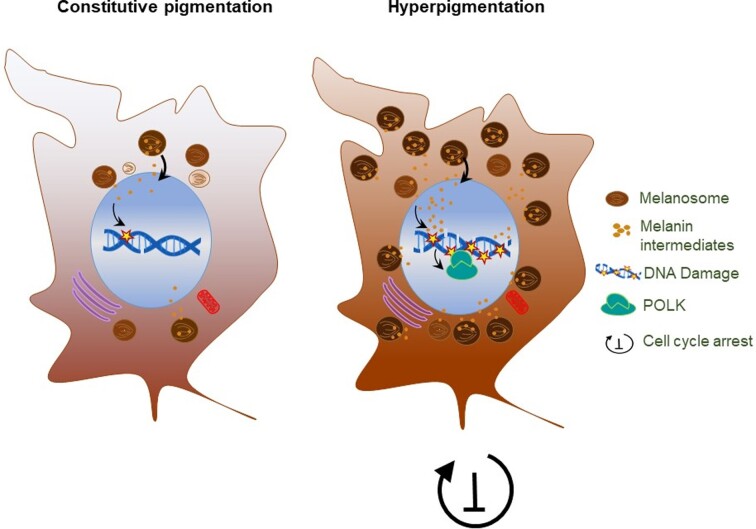

## INTRODUCTION

Skin pigmentation acts as an important barrier against penetration of harmful ultraviolet (UV) radiations. Melanin polymer with its broad absorption spectrum protects the genome from damage by high energy UV rays (1,2). Hence, skin tanning is an essential physiological response for protection against UV-induced mutagenesis and consequent risk of malignancy. The adaptive nature of pigmentation is evident in world-wide distribution of basal skin tone as well as the extent of tanning in populations residing across latitudes that differ in incident UV flux (3). The presence of melanin laden melanosomes in keratinocytes protects these cells from UV damage (4). However, synthesis of melanin within melanocytes poses challenge as the intermediates are highly reactive and potentially genotoxic (5–9); they also have a potential to induce melanoma and facilitate its progression (9–11). Melanin intermediates are also indicated as immunosuppressants during cancer therapy (12). Variety of factors contribute to the mutation burden in melanocytes, among these the role of melanogenesis, if any, remains obscure.

The nature of DNA damage caused by UV-A, UV-B and UV-C, as well as the cellular response are very well-characterized (13,14). UV-signature mutations arise in keratinocytes consequent to unrepaired DNA photoproducts, such as cyclobutane pyrimidine dimers (CPDs) and pyrimidine 6–4 pyrimidones (6–4 PPs). These are resolved via Nucleotide Excision Repair (NER) pathway. The autosomal recessive syndrome xeroderma pigmentosum caused by mutations in various genes in this pathway and the translesion polymerase eta (POLH), render individuals sensitive to sunlight (15,16). Tumor suppressor TP53 is the key orchestrator of UV-mediated DNA damage in keratinocytes, that induces *POMC* gene expression, resulting in α-MSH secretion (17). In neighboring melanocytes, α-MSH activates the downstream MC1R signaling. In addition to promoting melanogenic response, this pathway invokes NER which pre-emptively prepares melanocytes for DNA repair (18).

Genetic risk factor of melanoma links to the red hair phenotype of individuals with lighter skin tone due to the presence of pheomelanin. Systematic studies have elucidated the role of oxidative damage in UV-independent carcinogenesis, induced by pheomelanin (19). Epidemiological studies suggest that the highest melanoma risk is associated with intermittent exposure to UV rays (20). UV-induced free radicals excite electrons in melanin to create a quantum triplet state that transfer energy to DNA and initiate dark CPD formation. The work shows the transfer of energy to DNA, inducing the formation of CPDs (21). These lesions are considered responsible for the characteristic UV-signature C > T transitions. Hence, UV directly and in conjunction with melanin is likely to cause DNA lesions and initiate mutations. Additionally, as pheomelanin is capable of mutagenesis independent of UV, it is generally believed that formation of eumelanin is protective and hence its role in causing DNA damage is not systematically investigated. In a recent work, investigators identified that melanocytes contain genomic locations that are prone to modifications in several loci implicated in melanoma (22). We examined the role of melanin and its intermediates generated during sustained melanogenesis to modify DNA and elicit counteracting cellular response.

To investigate this, in the current study we employ a synchronized, temporally resolved pigmenting system using B16 melanoma cells that produce only eumelanin and confirm our results in primary human melanocytes. In a UV-independent manner, the rate of eumelanin production is further enhanced with substrate tyrosine, suppressed with pharmacological inhibition using Phenylthiourea (PTU) as well as CRISPR-based abrogation of melanin synthesis. Sustained eumelanin synthesis increases γH2AX foci and abasic sites formation in B16 melanoma cells. In response, translesion DNA polymerase K (Polκ) is induced through ATR-CHK1 signaling. Polκ is critical for the induction of cellular replication stress response and in the absence of Polκ, sustained melanogenesis results in continued proliferation of melanocytes with DNA damage that could lead to genome instability. Extracellular synthesis of melanin as well as treatment with DHI (dihydroxyindole), one of the intermediates of eumelanogenesis, causes DNA breaks and induces Polκ in B16 melanoma cells. Our study establishes that eumelanogenesis through melanin intermediates can produce lesions in DNA. These observations highlight the counteracting role of the translesion polymerase Polκ in combating melanin-induced DNA damage. Meta-analysis of TCGA-melanoma data shows elevated mutation burden in human melanoma patient samples with high Polκ mRNA levels and a concomitant decrease in survival. Thereby, a complex interplay between melanin-induced DNA damage, error prone nature of polymerase Polκ and induction of replication stress response shapes the DNA repair functions of melanocytes.

## MATERIALS AND METHODS

### Cell lines, reagents and media

B16 cells were obtained from ATCC. Primary human melanocytes (Normal Human Epidermal Melanocytes, NHEM) were obtained from Lonza. All cell culture reagents were obtained from GIBCO Life technologies. siRNAs and shRNA were purchased from Dharmacon (GE Healthcare) and transfections were performed using Dharmafect II for siRNAs and Lipofectamine 2000 for shRNAs. Midiprep plasmid preparation was performed using Qiagen Midi DNA kit. KAPA SYBR FAST qPCR Master Mix was obtained from KAPA Biosystems. Genomic DNA and RNA isolations were performed using Macherey Nagel NucleoSpin® TriPrep kit (Cat no.: 740966).

### Setting of low density based progressive pigmentation model of B16 mouse melanoma cells

Cultures were set up as described by (23). Briefly, depigmented B16 (called as day 0 cells) cells were seeded at a density of 100 cells/cm^2^ in DMEM high glucose media (Sigma; D5648) with 10% FBS (Invitrogen, 10270) to make them pigmented by day 6 to day 8. These cells, starting from day 4 until day 8 are the pigmented cells. During the span of pigmentation, the media was not replenished.

### Culturing and chemical treatments on melanocytes

B16 were cultured in DMEM high glucose and 10% FBS, and primary human melanocytes were grown in M254 (Gibco, M-254-500), both at 5% CO_2_ concentration. Efforts were diligently made to minimize the influence of ambient light throughout the culture and treatment processes. Special care was taken to ensure minimal exposure during passage and media change, limiting exposure to white light of fewer than 900 lx. This level of light intensity is at least three logarithmic orders lower than the energy required for photosensitization. All treatments were given at 24 h post seeding. 200 μM of Phenylthiourea (PTU, Sigma; P7629) was used for depigmentation, and 1 mM of l-tyrosine was used for hyperpigmentation of cells. 2 mM of hydroxyurea (Sigma; H8627) for 3 h was used for inducing replication stress, 5 J/m^2^ of UVA and 200 mJ/cm^2^ of UVB were used as photooxidative damaging agents. 100 mM H_2_O_2_ for 15 min was used as an oxidative stress inducer. AZ20 (Sigma; SML1328) was used as an ATR inhibitor at 50 nM concentration. PP242 (Medchem: HY-10474) was used as an mTOR inhibitor at 2 μM.

### Detection of DNA base modifications by ELISAs

Genomic DNA was isolated using the Macherey Nagel Nucleospin Triprep kit. Abasic sites and 8-oxoGuanine were determined using ELISA kits from cell biolabs (OxiSelect™ Oxidative DNA Damage Quantitation Kit, AP sites, STA-324 and OxiSelect™ Oxidative DNA Damage Quantitation Kit, 8-OHdG, STA-320) using manufacturer's instructions.

### NBT assay

Plasmid DNA was incubated either alone (control DNA), with substrate L-DOPA (DNA + DOPA) or with melanin synthesis reaction containing L-DOPA and enzyme tyrosinase (DNA + Mel). These samples, along with the melanin synthesis reaction without DNA (Mel only), or DNA added to the reaction just before column purification [DNA+(Mel)] were column purified after 16 h of incubation, to remove free melanin intermediates, enzyme and melanin from DNA. Hence, only DNA (modified or unmodified as mentioned above) is taken ahead. These samples are then quantified, and equal concentrations are incubated with NBT for 15 min at room temperature. Samples are then transferred in cuvette and absorbance at 570 nm (corrected at 700 nm) is taken for detection of formazan (formed by quinone-DNA adducts). The absorbance is converted to concentration of formazan using the Beer-lambert's law, *A*= ϵ*bC* (*A* = absorbance, *b* = path length of cuvette, i.e. 1 cm, *C* = concentration of formazan in μM and ϵ = molar absorption coefficient of formazan, i.e. 13 000 M^−1^ cm^−1^ at 570 nm) (24).

### Detection of reactive oxygen species and apoptotic cells

Relative levels of reactive oxygen species were determined using CM-H2DCFDA (2′-7′-dichlorodihydrofluorescein diacetate) assay kit (Thermo, Cat no. C6827). Detection of apoptotic cells was done using The Alexa Fluor® 488 annexin V/Dead Cell Apoptosis Kit (Invitrogen, V13241) as per manufacturer's protocol.

### Cell cycle analysis of B16 cells

B16 cells were trypsinised and washed once with PBS. Cell pellet of approximately 3–5 million cells was resuspended in 200–300 μl of 70% chilled ethanol and was stored overnight in minus 20°C. For analysis, cells were spun at 7000 rpm for 10 min at 4°C. Supernatant ethanol was discarded and washed thrice with 1ṣ PBS to remove traces of ethanol. Finally, cells were resuspended in 100–200 μl of (PBS + 0.1% Triton X100) along with RNase A (Sigma; R6513) to a working concentration of 0.5 mg/ml. These were incubated overnight at room temperature. After taking out some cells as unstained control, 2 μl of propidium iodide (2 mg/ml) was added to approximately 100 μl of cell suspension and incubated at room temperature for 10 min in the dark. Cells were then directly used for flow cytometric analysis.

### Alkaline and neutral comet assay

Corning glass slides were pre-coated with 0.1% LMA and dried at 55° for 1–2 h on a hot plate. Meanwhile, the cells were trypsinised and 30 000–50 000 cells from each set were taken in a 1.5 ml eppendorf tube and pelleted down by centrifugation. A single wash of 1× PBS was given for complete removal of media from cell pellet. The cells were uniformly resuspended in 50 μl of PBS and 550 μl of preheated 0.75% agarose was added onto cells, mixed well and layered on the precoated slides after removing them from heatblock. The slides were now allowed to solidify by keeping them at 4°C for 20 min. These were then immersed in chilled Alkaline Lysis buffer taken in a container while keeping the buffer on ice for another 20 min. Slides were gently taken out and rinsed thrice in chilled AMQ. While keeping the AMQ on ice, the slides were immersed from sides and taken out without giving any mechanical shock. Finally, the slides were dipped in Electrophoresis buffer (pH-13) and incubated for 40 min on ice. After this, 600 ml of electrophoresis buffer was added to the electrophoresis unit and the slides were immersed in it while aligning them straight in electric field. Electrophoresis was run at 24 V (2 V/cm), 400 mA for 20 min. For neutral comet assay, prepared slides were incubated in chilled Neutral electrophoresis buffer (pH 9) for 1 h, and then electrophoresed in the same buffer at 24 V (2 V/cm), 400 mA for 20 min. After completion of run, slides were taken out and rinsed thrice with chilled AMQ and dipped in chilled neutralization buffer (pH 7.4) for 5 min while keeping the buffer on ice. Finally, the slides were taken out and rinsed again in AMQ and kept on hot plate at 55°C for 3–4 h till complete drying. Slides were stained with 50 μM propidium iodide and imaged on Leica microscope. Analysis was performed by KOMET software.

For OGG-1 modified comet assay after the cell lysis, slides were incubated with OGG-1 buffer alone (-OGG-1) or with 0.5 μg/ml OGG-1 (+OGG-1) at 37°C in a humidifying chamber for a period of 45 min. The slides were then processed for alkaline comet assay, as mentioned above.

### RNA isolation, cDNA synthesis and real time PCR

RNA isolation was performed using Macherey Nagel triprep kit using manufacturer's instructions. 500 ng of RNA was reverse transcribed using Superscript III cDNA synthesis kit (Life Technologies) according to manufacturer's protocol. Gene expression analysis by quantitative real-time PCR was performed on a Roche Light Cycler 480 II real-time cycler using the KAPA SYBR FAST qPCR Master Mix (KAPA Biosystems Cat no. 740966) to evaluate transcriptional regulations. Gene specific primers were designed using NCBI Primer Blast tool (https://www.ncbi.nlm.nih.gov/tools/primer-blast/) and obtained from Sigma Aldrich.

**RT-PCR primers:** Details of all the primers used in the study are given in Supplementary Table S1.

### DNA fiber assay

Cells were treated with 25 mM CldU, and then with 250 mM IdU at 37°C for 20 min each. Subsequently, the cells were washed twice with PBS, trypsinized and adjusted to approximately 400 000 cells/ml for further processing. To perform DNA spreading, a 2 μl cell suspension was placed on the non-frosting side of a microscopic slide (Corning, Cat no. 2948-75x25) and allowed to dry for 5–7 min. Then, 7 μl of spreading buffer was added and incubated for 2 min. The slides were then tilted at an angle of 20–40°, so that the droplet moves to the bottom end of the slide at a constant speed. Slides were then fixed by 1 ml of methanol and incubating for 10 min. After denaturing with 2.5 M HCl for 1 h, the samples were subjected to immunostaining with rat monoclonal anti-BrdU and mouse monoclonal anti-BrdU primary antibodies for CldU and IdU respectively. Slides were imaged using Leica confocal microscopy, and LAS software (Leica) was used to quantify the replication fork length across the samples.

### Cell fractionation and western blot analysis

For whole cell lysate B16 cell pellet was resuspended in NP40 Lysis Buffer (Thermo; ALF-J60766-AP) reconstituted with protease and phosphatase inhibitors (750 μl of NP40 lysis buffer, 1× PIC, 10 mM sodium pyrophosphate, 1 mM sodium orthovanadate, 10 mM sodium glycerophosphate, 1 mM PMSF). Cells were incubated in lysis buffer overnight at −80°C and centrifuged at 13 000 rpm for 20 min at 4°C, protein supernatant was collected and estimated using standard BCA protocol (Pierce BCA protein assay kit; Thermo). Cellular fractionation was performed by resuspending the cell pellet in hypotonic Buffer A (10 mM HEPES, pH 7.9, 10 mM KCl, 1.5 mM MgCl_2_ and 0.5 mM DTT) while keeping the pellet on ice and incubating for 5 min. Cells were further lysed using strokes of a hand-held homogenizer. When >70% of cellular lysis was achieved as visualized under 40× microscope, suspension was centrifuged at 1000 rpm for 5 min at 4°C. Supernatant (cytosolic fraction) was separated and nuclear pellet was processed similar to whole cell pellet. 30 μg of the protein was used for proteins in the size range of 20–150 kDa. Primary antibody incubations were done for overnight in cold room (4°C). Subsequent to incubation with the primary antibody, the membrane was washed thrice with TBST containing 0.1% tween and were further incubated with corresponding HRP conjugated secondary antibody at 1:5000 dilution in 5% BSA 5% Skim milk or for one hour at room temperature. Membrane was washed in TBST with 0.1% Tween-20, thrice for 15 min each and developed using ECL reagent.

### Antibodies

Following primary antibodies were used. Polκ (ab57070), γH2AX (CST 9718), total H2AX (CST 2595), pChk1 (CST 2348P), Total Chk1 (Invitrogen PA512096), pATR (CST 2853), total ATR (CST 2790), p53 (ab38497), p21 (CST 9706), PCNA (SC56), DCT (ab74073), Tyrosinase (custom synthesized genscript), mouse anti-BrdU antibody (ab136650) and Rat anti-BrdU antibody (ab6326). Secondary antibodies conjugated to Alexa fluor for immunofluorescence were procured from Life technologies (USA). Secondary antibodies conjugated to HRP required for western blot were obtained from GE Healthcare.

### Immunocytochemistry on B16 cells

Cells were seeded on UV treated sterile coverslips (Corning) in six well plates. Cells were fixed with 2% paraformaldehyde in PBS for 10 min at 37°C. Further, they were permeabilized with 0.1% Triton® X-100 in PBS for 15 min at room temperature on slow orbital shaking. After three consecutive washes with 1× PBS, cells were blocked with 5% NGS (Jackson's immunoresearch) for 1 hour at room temperature on slow orbital shaking. Cells were given a single wash of PBS and incubated with γH2AX antibody (CST 9718, 1:200) or anti Polκ (ab57070, 1:100 in 1% NGS) for 2 h at room temperature. Post primary antibody incubation cells were washed thrice with PBST (PBS + 0.1% Triton X-100) for 5 min each on slow orbital rotation and then incubated in secondary Alexa Flour 568 anti-mouse antibody (1:500 in 1% NGS) for 1 h at room temperature in dark. Finally, cells were washed thrice with IX PBST and mounted on glass slides with 10 μl of antifade DAPI and sealed the coverslips using acetone. Imaging was done using Leica confocal microscopy and quantitated using LAS software (Leica). Average nuclear fluorescence intensity was determined using Leica Application suite AF (LAS AF) software.

### Generation of tyrosinase mutant B16 cells

Tyr CRISPR was designed using ECRISP (http://www.e-crisp.org/E-CRISP/). After overlap PCR of the CRISPR, it was *in vitro* transcribed using mMessage mMachine T7 ULTRA kit (Thermo; AM1345). For transfections, B16 melanoma cells were seeded at a regular density of 2 × 10^5^ cells per well of a six well plate. A complex of CRISPR RNA with Cas 9 protein was prepared and incubated for 20 min at room temperature. 500 μl of optiMEM (gibco; 31985070) along with 3 μl of Lipofectamine 2000 (Invitrogen; 11668019) was added to the above mix and incubated again for 20 min. After incubation, media was removed from culture and given a single wash of 1× DPBS. CRISPR mix was added to these cells after making up the volume to 1 ml with fresh optiMEM. This was incubated for 6 h after which media was changed to terminate transfections.

These transfected cells were used for setting up multiple LD culture flask in T75. The depigmented colonies arising from a single cell were manually picked up by visual selection under microscope followed by *in situ* trypsinization on Day 7 of LD culture. These colonies were expanded and analyzed for tyrosinase levels and activity. Control for this is a normally pigmenting unedited clone from the same experiment.

### siRNA transfections in B16 melanoma cells

B16 cells were seeded in 6-well plates at a density of 2 × 10^5^ cells per well. Transfections were carried out with Dharmafect II reagent as per manufacture's protocol. After 48 h, the cells were trypsinised and pelleted for RNA or protein isolations. For low density cultures (LD), cells were transfected on Day 5 of cycle and terminated on Day 7. During transfections, culture media was stored in sterile falcon or flask. After transfections, cultures were given a single wash of DPBS and replaced back with same media for cells to normally pigment during the course of LD cycle.

### shRNA transfections and stable knockdown of polκ in B16 melanoma cells

All shRNA’s (GIPZ Lentiviral constructs) were purchased from Dharmacon (GE Healthcare). Polκ shRNA (RMM4532-EG27015) glycerol stocks were used for generation of stable cell lines for Polκ knockdown. B16 cells were transfected with Polκ silencing plasmid. 24 h post transfections, selection of stable cells was done by treatment with Puromycin. Cells were kept under selection pressure for 3–4 weeks by regular change in media with fresh addition of puromycin every alternate day. In case of confluency, cultures were passaged, with treatment starting at 24 h post seeding. This was done until all the cells showed consistent GFP fluorescence.

### *In vivo* model of melanoma induction in mice

All animal procedures were done in accordance with the Indian Committee for the Purpose of Control and Supervision of Experiments on Animals (CPCSEA), (Section 15(1) of the Prevention of Cruelty to Animals Act, 1960; Registration no. 38/GO/Re Bi/SL/99/CPCSEA). Institutional Animal Ethics Committee approved all experiments and procedures carried out on the animals. For induction of melanoma tumors, Polκ knockdown stables or the non-silencing control cells were injected in 5 outbred male/female C57BL/6 mice each at 4–6 weeks of age (National Institute of Immunology, Delhi). A single subcutaneous administration of 10^6^ cells in 100 μl volume of cell suspension in right hand side flank region was given. The time and appearance of the first tumor (latency period) as well as the number of mice with tumors (incidence) was recorded during the study. At different time intervals i.e. on Day 11, Day 14 and Day 17, tumour was excised; length and width were measured using a Vernier Caliper.

### Statistical analysis and graphs

GraphPad prism was used to plot the graphs. Statistical analysis to obtain significance in the data was performed using Student's *t*‐test, one-way ANOVA or two-way ANOVA. Adjusted *P*-value (*P*) > 0.05 is marked as ns, ≤ 0.05 is marked as *, *P* ≤ 0.005 as **, *P* ≤ 0.0005 as *** and *P* ≤ 0.00001 as ****.

## RESULTS

### Cell-based model to study the effect of progressive pigmentation

To systematically investigate the effect of melanogenesis on DNA integrity, we resorted to the use of B16 progressive pigmentation model that permits the kinetic study of pigmentation and associated cellular changes in melanocytes (23). Choice of B16 cells that synthesize purely eumelanin, further enabled segregating the role of pheomelanin (25). In this model, B16 cells are grown at a high cell density of >10 000 cells/cm^2^ which results in depigmented melanocytes. To induce pigmentation, cells are plated at a low cell density of 100 cells/cm^2^. These cells progressively pigment over a course of 7 days. Thereby, cells on day 0 are depigmented, days 4–5 have intermediate pigmentation and days 6–7 cells are completely pigmented, allowing for progressive changes to be studied. To further perturb the pigmentation, in this study we compare the basal pigmentation state with hyperpigmentation induced by the treatment of 1 mM l-tyrosine (Tyr) that serves as a substrate for tyrosinase enzyme and augments melanogenesis (Supplementary Figure S1A). By the pharmacological inhibition of Tyrosinase with 200 μM phenylthiourea (PTU), we achieve decreased pigmentation in this progressive pigmentation model.

### Melanogenesis causes DNA damage

**Figure 1.**
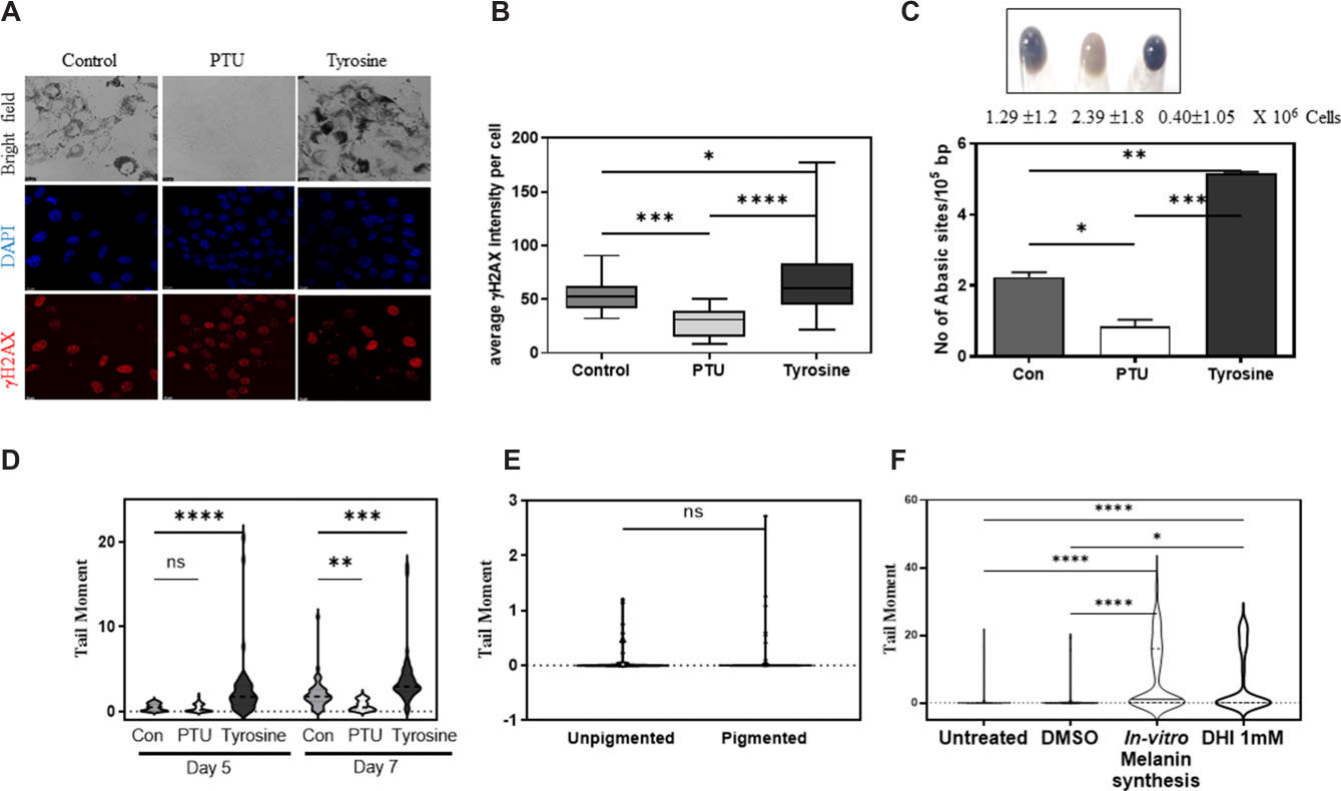
Melanin synthesis causes γH2AX foci formation DNA strand breaks and abasic sites formation. (**A**) Immunofluorescence of B16 cells treated with PTU and tyrosine with phosphorylated H2AX antibody. Nuclear DNA stained with DAPI (blue) and γH2AX in (red). Experiment was performed with two biological replicates and a representative image is depicted. Scale bar 10 μm. (**B**) Quantitation of mean fluorescence intensity per cell of γH2AX from two biological replicates of pigmented day 7 PTU or tyrosine treated cells (shown in A). Data represented as a box plot, horizontal line represents mean and whiskers represent SEM. Ordinary one-way ANOVA was performed for multiple comparisons. Adjusted *P*-value: * *P*-value < 0.05, ***P-value < 0.001, **** *P*-value < 0.0001. (**C**) (Top) Cell pellet of day 7 B16 mouse melanoma cells grown at low density (100 cells/cm^2^). Cells were left untreated for control treated with tyrosinase inhibitor 200 μM phenylthiourea (PTU) or 1mM tyrosinase substrate L-tyrosine (Tyr) for 7 days. Number of cells, mean ± SEM across three biological replicates is depicted below the image of the cell pellet. Numbers represent mean ± SEM cell counts across biological triplicates. (Bottom) Number of abasic sites in the genomic DNA was estimated by an aldehyde specific conjugation of biotin and subsequent detection using streptavidin based detection. Using standards, abasic sites per 10^5^ bp is estimated. Bars represent mean ± SEM across duplicate biological experiments, each conducted in triplicates. Ordinary one-way ANOVA was performed for multiple comparisons * *P*-value < 0.05, ** *P*-value < 0.01, *** *P*-value < 0.001. (**D**) Single cell electrophoresis followed by comet analysis of B16 cells undergoing varying levels of pigmentation in the presence of PTU and tyrosine (alkaline comet assay). Experiment was carried out at mid phase (day 5) and late phase (day 7) of pigmentation. Mean tail moment distribution across each population of duplicate biological experiments with atleast 50 comets analyzed is depicted by a violin plot. Two-way ANOVA was performed. Adjusted *P* values; ns non-significant, * *P*-value < 0.05, ** *P*-value < 0.01, *** *P*-value < 0.001, **** *P*-value < 0.0001. (**E**) Neutral comet assay on B16 unpigmented (day 0) and pigmented (day 7) cells. Mean tail moment distribution across each population of duplicate biological experiments with atleast 50 comets analyzed is depicted by a violin plot. Student's unpaired t-test was performed. *P* values ns non-significant. (**F**) Single cell electrophoresis followed by comet analysis of B16 cells untreated, treated with DMSO for 24 h, melanin synthesis (*ex-cellulo* L-tyrosine and tyrosinase added to cell media) for 24 h or cells treated with 1 mM dihydroxyindole (DHI) for 24 h (alkaline comet assay). Mean tail moment distribution across each population of duplicate biological experiments with atleast 50 comets analyzed is depicted by a violin plot. Ordinary one-way ANOVA was performed. Adjusted *P* values: * *P*-value < 0.05, **** *P*-value < 0.00001.

Melanin contains free radicals, and during its biosynthesis several reactive quinone intermediates are generated that can pass the limiting melanosome membrane and result in damage (9,26). By cytometric DCFDA staining of differentially pigmented cells, we observed the highest DCFDA mean fluorescence intensity in tyrosine treated hyperpigmented cells and least in PTU treated cells in which melanin synthesis is minimal (Supplementary Figure S1). We surmised that these free radicals could cause cellular DNA damage. Nuclear γH2AX foci that are associated with DNA damage and strand breaks, increased with tyrosine treatment in pigmented day 6 cells and pre-treatment with the melanogenesis inhibitor PTU, reduced the foci formation (Figure 1A, B). Furthermore, abasic sites detected by direct ELISA in the genomic DNA followed the same pattern as cellular free radical levels (Figure 1C). However, DNA base oxidation as detected by 8-hydroxy-2-deoxyguanosine (8-OHdG) levels were comparable across the three pigmentation groups (Supplementary Figure S1C). Further, we performed quantitative real-time PCR on genomic DNA obtained from differentially pigmented cells after subjecting the DNA to 8-oxoguanine DNA glycosylase-1 (OGG-1) (Supplementary Figure S1D). This was confirmed using OGG-1 modified comet assay (Supplementary Figure S1E). In these two assays, we did not observe significant changes induced by OGG-1 treatment in the differentially pigmented samples, possibly indicating that the DNA integrity altered by pigmentation is not a direct oxidative DNA damage.

Surprisingly not only the γH2AX phosphorylation was elevated with tyrosine treatment and decreased with PTU, the total H2AX levels also followed a similar trend (Supplementary Figure S1F). In alkaline comet assay, the level of associated DNA nicks was high in day 7 pigmented cells, tyrosine treated hyperpigmented cells demonstrated maximal DNA nicks. The least tail moment was observed in the PTU treated depigmented cells (Figure 1D & Supplementary Figure S1G). The mean tail moment progressively increased in control and tyrosine treated cells on day 7, compared to day 5 as the melanin content increased. We then proceeded to assess DNA breaks by neutral comet assay. In the neutral comet assay we observed very little breaks and the tail moment was close to 1–2 units in both pigmented and unpigmented cells, and did not observe significant differences between these two states (Figure 1E).

In all, these experiments suggest that ongoing melanogenesis is associated with DNA damage. On a closer look the following points emerge. Melanogenesis causes free radical generation but does not increase 8-OHdG levels in the cells. Instead, there is a marked elevation in abasic sites, which is clearly dependent on pigmentation. The increased intensity of γH2AX puncta classically associated with double stranded breaks, could also mark single stranded DNA regions as well as other DNA lesions (27,28). Since the neutral comet assay does not show comet formation upon pigmentation, double stranded breaks would not account for the observed DNA breaks. However, the alkaline comet assay increases the tail moment, progressively and predictably in a pigmentation dependent manner (Figure 1D). It is interesting to note that the abasic sites are prone to strand cleavage and result in enhanced comets as seen in alkaline comet assay (29). Therefore, the elevated tail moment observed in alkaline comet assay could be contributed by the abasic sites (alkali labile) as well as single stranded DNA lesions, if any, generated by melanin-based damage and the relative contribution is difficult to assess at this stage.

Finally, to confirm the role of melanin intermediates in causing the DNA damage, we subjected depigmented B16 cells to 1mM Dihydroxy Indole (DHI), one of the intermediates of eumelanin synthesis reaction, as well as exogenously increased melanin synthesis through *ex-cello* synthesis of melanin (with the substrate L-DOPA and enzyme Tyrosinase) added in the culture medium and performed alkaline comet assay. We observed an increase in the comet tail length with both the treatments, confirming that melanin intermediates can cause DNA damage (Figure 1F). The DNA damage due to melanin intermediates was known (5–9). By the systematic investigation of melanogenesis induction and its pharmacological perturbation, we unequivocally demonstrate the DNA damage using this cell-based model system.

Since melanin is a complex polymer, we could not demonstrate direct evidence for covalent conjugation of melanin intermediates to DNA. However, plasmid DNA incubated with melanin synthesis reaction (which was further purified to remove unbound melanin intermediates) demonstrated quinone-DNA adduct formation using coupled Nitro-blue tetrazolium (NBT) based detection (Supplementary Figure S1H). In support, DNA incubated with melanin intermediates during melanin synthesis showed altered mobility in agarose gel, while the same was not observed when the DNA is incubated with pre-synthesized melanin (Supplementary Figure S1I).

### Melanogenesis induced DNA alterations elicit a replication stress response

**Figure 2.**
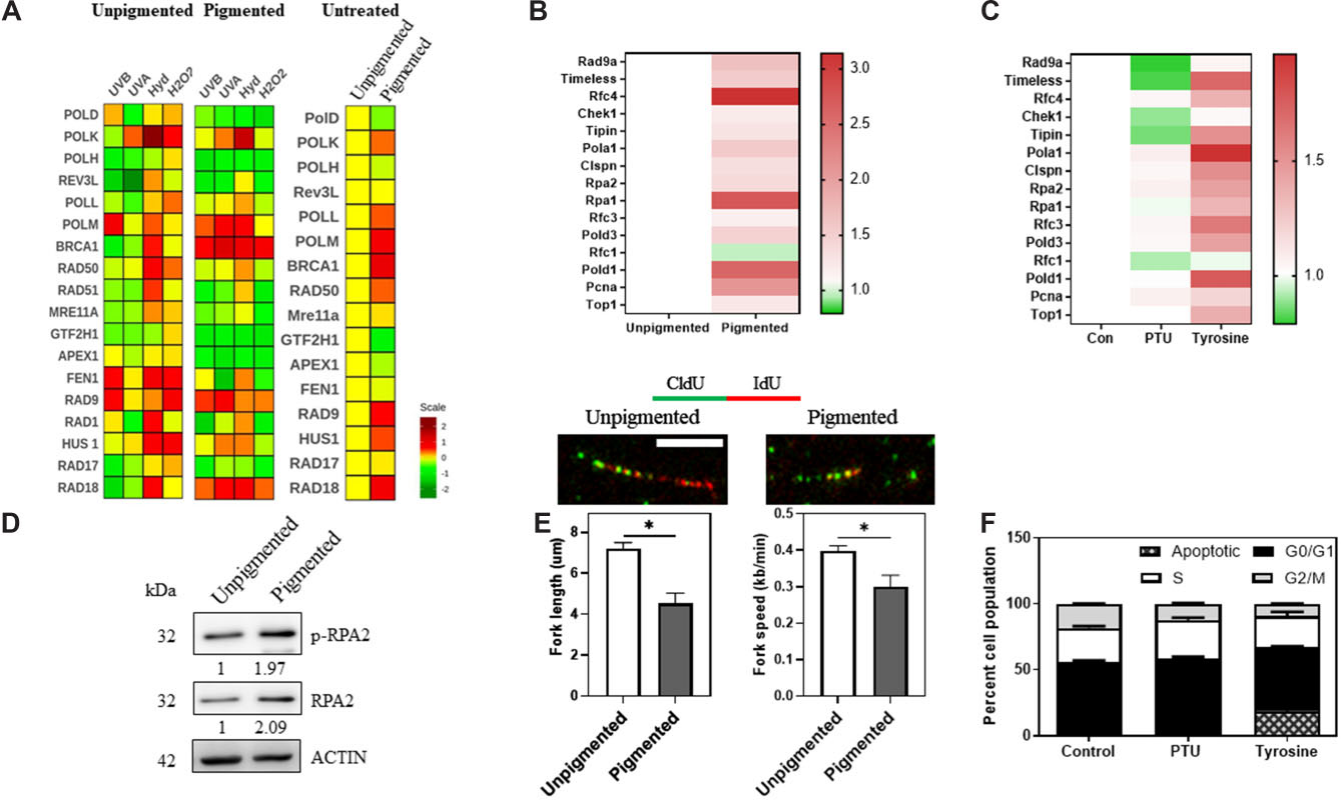
Melanogenesis induces replication stress response. (**A**) Fold change in mRNA levels of a panel of DNA repair genes by qRT-PCR analysis. The heat map represents fold change and compares DNA repair gene signature of depigmented (left), pigmented (middle) B16 cells to various DNA damaging agents. Untreated pigmented cells compared to depigmented control is depicted as a heat map (right). (**B**) Heat map of expression changes (fold change) in mRNA levels of a panel of known ‘replication stress response’ genes by qRT-PCR analysis in B16 melanoma unpigmented day 0 cells compared to pigmented day 7 cells across at least two biological replicates. (**C**) Heat map of expression changes (fold change) in mRNA levels of a panel of known DNA replication stress response genes by qRT-PCR analysis in B16 melanoma pigmented day 7 cells treated with PTU or tyrosine across three biological replicates. (**D**) Western blot images and quantitation of p-RPA2 and total RPA2 in B16 melanoma unpigmented day 0 cells compared to pigmented day 7 cells. Numbers below represent beta-actin normalized fold changes *wrt* shNT at day 0. Two biological replicates were performed. (**E**) DNA fiber assay of unpigmented day 0 and pigmented day 7 B16 mouse melanoma cells. (Top) Representative images of the DNA fiber tracts are shown. (Bottom) Quantification of fork length (μm) and fork speed (in kb per minute) is shown as bar graphs. Bars represent mean ± SEM across triplicate biological experiments. Student's unpaired *t*-test was performed. * *P*-value < 0.05. Scale bar 5 μm. (**F**) Cell cycle analysis carried out using propidium iodide staining detected by FACS is depicted as stacked bars. Sub G0 population is marked as apoptotic. Experiment was carried out in biological triplicates and mean ± SEM is depicted. Two-way ANOVA was performed. Adjusted *P* value: *P-value < 0.05 across comparison of G0/G1 population.

Having observed that melanogenesis causes DNA damage, we then proceeded to decipher the nature of response used to combat this unique challenge faced by melanocytes. Towards this we treated either unpigmented day 0 or pigmented day 7 B16 cells with known DNA damaging agents. UVA and UVB to assess photo-oxidative damage, hydroxyurea for replication stress and H_2_O_2_ for oxidative damage were employed. Pigmented and unpigmented cells that were not treated with any DNA damaging agents were included, and the expression of a panel of DNA repair genes were analyzed by qRT-PCR analysis after 24 h treatment. Signature pattern of expression for each of the damage was clearly distinct, and notably the pattern of gene expression induced by pigmentation closely paralleled that of hydroxyurea treatment that results in replication stress (Figure 2A).

Molecularly, replication stress is slowing or stalling of replication fork progression associated with DNA replication (30). Although the importance of replication stress response is well-recognized, molecular events associated with this remain enigmatic. During progressive pigmentation using our previous microarray data, we observed that an expanded battery of known ‘replication stress response genes’ was found to be elevated (Supplementary Figure S2A) (31,32). Validation of this set of genes in depigmented (day 0) and pigmented (day 7) B16 cells confirmed that these genes were induced during pigmentation (Figure 2B). Expression of these genes was further augmented with tyrosine and reduced with PTU treatment confirming elicitation of replication stress response during pigmentation (Figure 2C). Protein levels as well as the phosphorylation status of RPA2 at Ser 33 residue that are hallmarks of replication stress response also showed a pigmentation dependent induction (Figure 2D). Based on DNA fiber assay, we further demonstrate decreased fork length and fork speed (Figure 2E).

When compared to control pigmented cells, a consistent reduction in the number of cells on day 7 upon hyper-pigmentation with tyrosine, and an increase upon depigmentation with PTU was observed (Figure 1C, top). Cell cycle analysis using propidium iodide (PI) staining followed by flow cytometry to quantify cells in different phases of the cell cycle revealed that hyperpigmented cells have reduced S + G2/M population confirming a reduction in proliferation (Figure 2F). Tyrosine treatment not only reduced S + G2/M population, but it also resulted in a sizeable population of sub-G0 cells suggesting apoptosis. Annexin V- PI based apoptosis assay confirmed that higher uncontrolled hyperpigmentation results in an increase in the apoptotic population. While this cell death was seen to be higher in tyrosine treated cells which are hyperpigmented, control pigmented cells also showed apoptosis compared to depigmented cells (Supplementary Figure S2C, D). Taken together we conclude that melanin causes DNA damage, and a key response is elicitation of replication stress resulting in the arrest of cell cycle progression.

### In response to melanogenesis, translesion polymerase polκ is induced by ATR-CHK1 pathway

**Figure 3.**
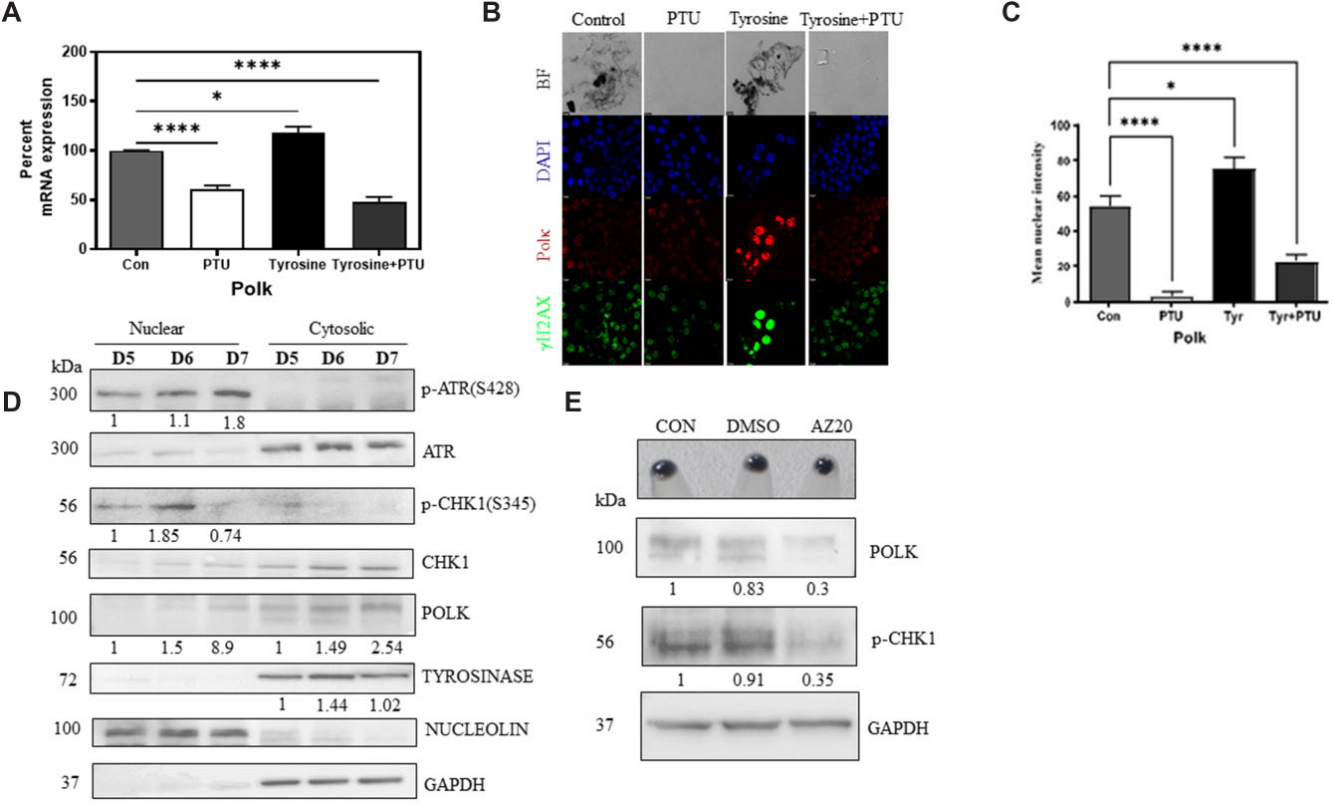
Melanogenesis induces Polκ via ATR-CHK1 signaling axis. (**A**) mRNA levels of *Polk* in B16 cells that are allowed to pigment differentially in the presence of 200 μM PTU, 1 mM tyrosine or both. Bars represent percent mRNA levels compared to control as mean ± SEM across five biological replicates. Ordinary one-way ANOVA was performed. Adjusted *P* values: * *P*-value < 0.05, **** *P*-value < 0.00001. (**B**) Immunofluorescence of B16 cells treated with 200 μM PTU, 1 mM tyrosine or both. Top panel is the bright field (BF) and dark granules are the pigment accumulation. Nuclear DNA stained with DAPI (blue), Polκ in red and γH2AX in (green). Scale bar 10 μm. Experiments were performed in triplicates. (**C**) Quantitation of mean nuclear fluorescence intensity of Polκ of differentially pigmented day 7 B16 cells treated with PTU, tyrosine or both (shown in B). Bars represent mean ± SEM across two biological replicates. Ordinary one-way ANOVA was performed. Adjusted *P* values: * *P*-value < 0.05, **P-value < 0.005, **** *P*-value < 0.00001. (**D**) Western blot analysis of nuclear and the post-nuclear (cytoplasmic) lysates of B16 cells on day 5, day 6 and day 7 of pigment accumulation. Numbers below the blot correspond to day 5 normalized expression of the indicated protein. Experiment was performed in duplicates. (**E**) Western blot analysis of B16 cells treated with DMSO or 50 nM AZ20, a selective inhibitor of ATR kinase. Numbers below the blot correspond to control normalized expression of the indicated protein *wrt* GAPDH. Experiments were performed in duplicates.

Response and repair mechanisms would be operational in the pigmented cell to sense and correct the DNA damage. Cells use specialized polymerases to resume DNA synthesis by bypassing it. For achieving this a battery of translesion polymerases are known to play an important role (33). We therefore studied the expression changes of all the DNA translesion polymerases during pigmentation in B16 melanoma cells. Profiling of expression changes using the earlier microarray data, in DNA translesion polymerases on different days of progressive pigmentation revealed that three of the translesion polymerases *Polk*, *Polh* and *Rev3l* were increased in expression at early and late-phase of pigmentation (Supplementary Figure S3A). To investigate whether their regulation is critically dependent on pigmentation, B16 cells were treated with PTU, tyrosine or both and on day 7 were subject to qRT-PCR analysis for these three candidate genes. Regulation of *Polk* paralleled the observed pigmentation (Figure 3A). Whereas, the other two translesion polymerases *Polh* and *Rev3l* were minimally altered and did not show an expected trend in their regulation with pigmentation and its suppression with PTU (Supplementary Figure S3B, C). Thereby, Polκ emerged as a promising candidate that could have an important role in DNA damage response during pigmentation.

Immunocytochemical localization of Polκ and labelling by γH2AX puncta was simultaneously carried out in differentially pigmenting B16 cells. We observed high Polκ as well as γH2AX labelling in tyrosine treated hyperpigmented cells (Figure 3B, C). Prevention of pigmentation by PTU reversed the levels of both Polκ and γH2AX staining, substantiating the induction to be pigmentation dependent. Whole cell lysate confirmed the induction of Polκ with progressive pigmentation (Supplementary Figure S3D). Subsequent cell fractionation followed by western blot analysis on different days of pigmentation confirmed induction of Polκ in the progressive pigmentation model (Figure 3D). From these lysates, a clear induction of nuclear localized phosphorylated ATR and phosphorylated CHK1 during pigmentation further indicated activation of DNA repair response pathway during progressive pigmentation.

Having observed an induction of Polκ during pigmentation and a concomitant increase in the ATR-CHK1 signaling axis, we hypothesized that the ATR-CHK1 pathway could be responsible for Polκ induction. To test this, during pigmentation, we treated the cells with 50 nM of ATR inhibitor AZ20. Western blot analysis confirmed decrease in p-CHK1 levels and reduction in Polκ (Figure 3E). Hence we identify Polκ induction to be under the control of ATR-CHK1 signaling axis. Thereby, this translesional polymerase is likely to function as a key sensor or a responder in combating the pigmentation induced DNA damage.

### Genetic ablation of tyrosinase gene curtails melanogenesis and the consequent DNA damage

It is likely that treatment with chemicals such as tyrosine and PTU could trigger the changes in Polκ levels in cells independent of pigmentation. Hence, we genetically ablated Tyrosinase, the key enzyme involved in melanin synthesis using CRISPR based methodology (Supplementary Figure S4A). The strategy involved transfection of high density unpigmented B16 cells with synthesized single guide RNA (sgRNA) against tyrosinase (Tyr) gene and Cas9 protein complex and plating them at a low density (100 cells/cm^2^) to initiate pigmentation. Individual depigmented and control pigmented colonies derived from a single cell, were then trypsinized and screened for their ability to pigment, TYR protein levels and *in vitro* enzyme activity involving L-DOPA zymography (Supplementary Figure S4B, C). Sequence confirmation identified a single base deletion downstream to the sgRNA sequence, resulting in a frameshift mutation (Tyr^fs118^) encoding a truncated protein (Supplementary Figure S4A). Thereby these mutant cells were compromised in their ability to make melanin and could be compared with B16 WT cells for studying DNA damage under conditions of low-density culturing wherein progressive pigmentation is induced.

**Figure 4.**
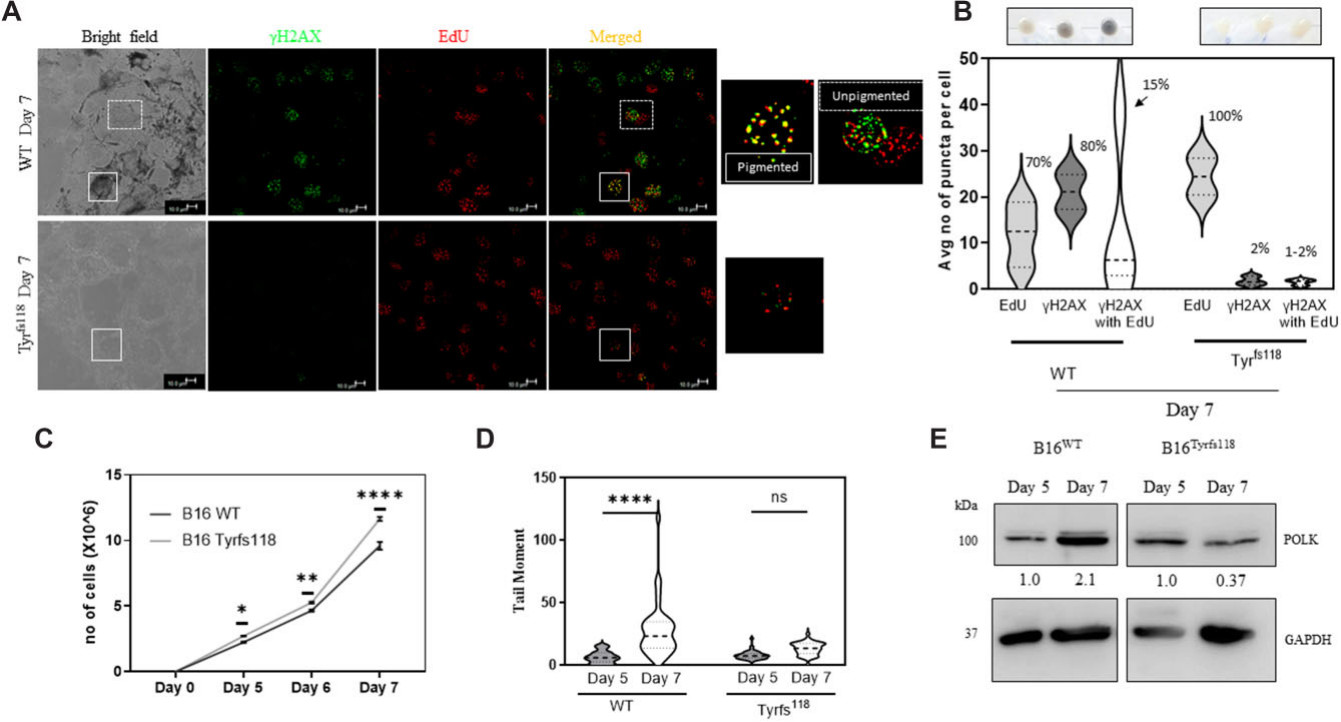
CRISPR-based genetic ablation of tyrosinase prevents melanogenesis, reduces DNA breaks and curtails Polκ induction. (**A**) Immunofluorescence images of B16 WT and B16 Tyrosinase mutant (Tyr^fs118^) cells that were allowed to pigment for 7 days. Bright field images indicate the presence of melanin granules. Labelling using 5-ethynyl-2′-deoxyuridine (EdU) (red) and γH2AX antibody (green). Merged images show co-localization of γH2AX with EdU (yellow). A zoomed in view of a single cell is shown as an inset. Scale bars represent 10 μm. (**B**) (Top) Cell pellets of B16 WT and Tyrosinase mutant (Tyr^fs118^) cells on days 5, 6 and 7 of pigmentation. (Bottom) Quantitation of average of the number of γH2AX puncta that are single positive or double positive (colocalizing with EdU puncta) per cell across 50 cells in B16 WT and B16 Tyr^fs118^ cells is depicted as a violin plot across three biological replicates. The percent population of cells with indicated puncta is mentioned alongside. (**C**) Growth curve analysis of B16 WT and Tyr^fs118^ cells on days 0, 5, 6 and 7 of pigmentation. Each point represents mean ± SEM across biological triplicates. Two-way ANOVA was performed. Adjusted *P* values: * *P*-value < 0.05, *** *P*-value < 0.001. (**D**) B16 WT and Tyr^fs118^ cells on days 5 and 7 of pigmentation were subjected to single cell electrophoresis and alkaline comet assay was performed. Mean tail moment distribution across each population of duplicate biological experiments with at least 50 comets analyzed is depicted by a violin plot. Two-way ANOVA was performed. Adjusted *P* values ns non-significant: **** *P*-value < 0.0001. (**E**) Western blot analysis of whole-cell lysates of B16 WT and B16 Tyr^fs118^ cells on day 5 and day 7 stages of pigmentation. Numbers below the blot correspond to day 5 GAPDH normalized expression of the indicated protein. Experiments were performed in biological duplicates with similar results.

We first assessed the staining of day 7 B16 WT and Tyr^fs118^ cells with γH2AX foci and detection of newly synthesized DNA using EdU coupled fluorophore labelling (Figure 4A). B16 WT pigmented cells have a significant overlap of EdU with γH2AX signals indicating that these are regions of DNA damage where new DNA synthesis is ongoing. Interestingly, these wild type cells showed heterogeneity in double stained population. On closer analysis, the heavily pigmented cells had several double positive foci, whereas cells with moderate pigmentation had lower levels of colocalized puncta (Figure 4B). Analysis of the EdU and γH2AX colocalized spots clearly suggested that the pigmented cells had a higher proportion of DNA breaks, that are in the process of repair wherein fresh DNA synthesis is ongoing. The proportion of these cells was around 15% of the total population, indicating a possible translesion synthesis in only the minority of heavily pigmented cells. Whereas B16 Tyr^fs118^ remain depigmented and had lower level of γH2AX staining concomitant to a high EdU puncta per cell. This suggested higher proliferation, which was confirmed by the growth curve analysis of B16 WT and B16 Tyr^fs118^ cells (Figure 4C). DNA breaks determined by comet analysis indicated that the mutant depigmented cells had minimal DNA damage, as inferred from alkaline comet assay (Figure 4D). Furthermore, western blot analysis showed sustained induction of Polκ only in B16 WT pigmented cells but not in the depigmented B16 Tyr^fs118^ cells, in which the levels decreased from day 5 to day 7 (Figure 4E). Thereby the overall DNA damaging effects of melanin and the cellular response of melanocytes by invoking Polκ is confirmed using this genetic model. While it is tempting to speculate that the difference in proliferation is due to the lack of sustained Polκ induction in Tyr^fs118^ cells, it cannot be unequivocally concluded. This establishes the causal link between melanogenesis, DNA damage and Polκ during pigmentation.

### Pigmentation induced DNA breaks and elicitation of polκ response is recapitulated in primary human epidermal melanocytes

**Figure 5.**
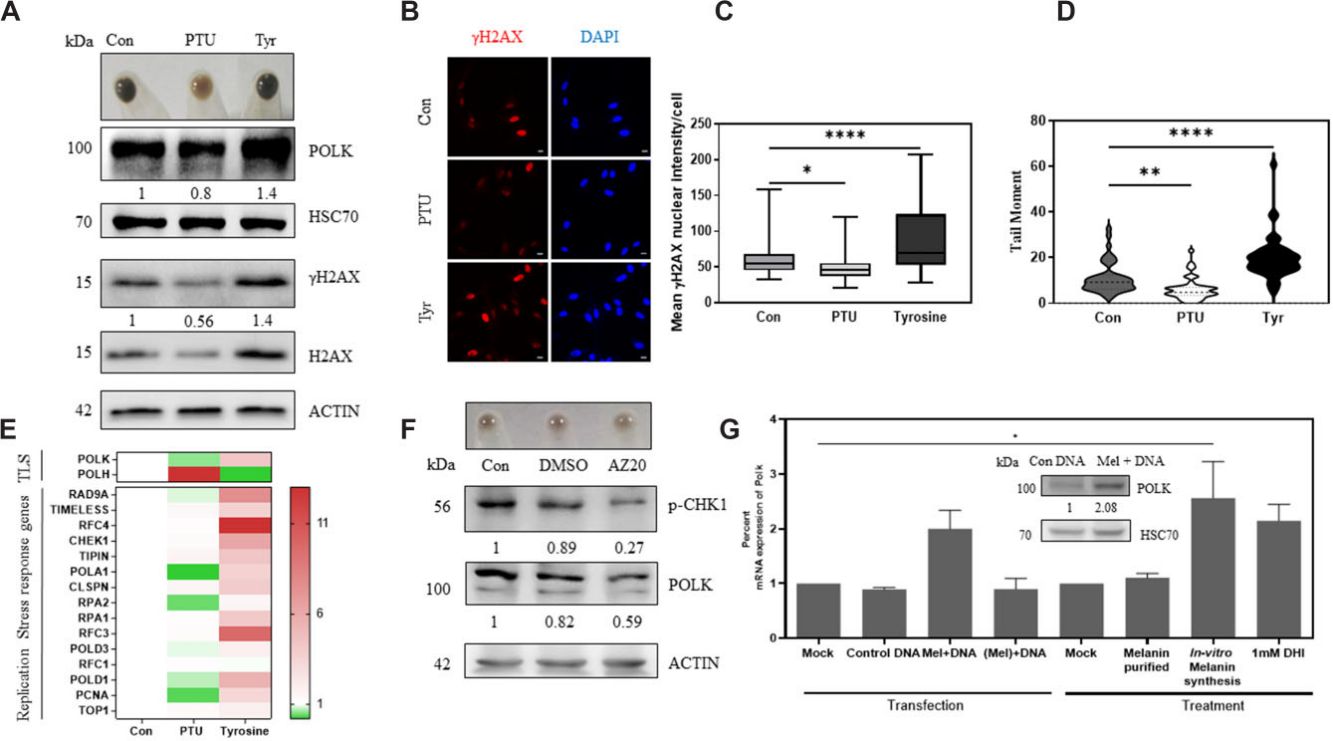
Normal human epidermal melanocytes (NHEM) respond to pigmentation induced DNA breaks by elevating Polκ. (**A**) NHEM cells were treated with 200 μM PTU or 1mM tyrosine for 7 days for differential pigmentation. (Top) Cell pellet, (bottom) western blot analysis of cell lysates with POLK, HSC70, phosphorylated H2AX, total H2AX and beta actin antibodies. Numbers below the blot correspond to control normalized expression of the indicated protein. Experiments were performed in biological duplicates. (**B**) Immunofluorescence of NHEM treated with PTU or tyrosine with phosphorylated H2AX antibody. Nuclear DNA stained with DAPI (blue) and γH2AX in (red). Experiments were performed with two biological replicates. Scale bar 10 μm. (**C**) Quantitation of mean fluorescence intensity per cell of γH2AX from two biological replicates of NHEM treated with PTU or tyrosine (shown in B). Ordinary one-way ANOVA was performed for multiple comparisons. Adjusted *P* values: * *P*-value < 0.05, **** *P*-value < 0.0001. (**D**) PTU and tyrosine treated NHEM cells were subjected to single cell electrophoresis and comet analysis (alkaline comet assay). Mean tail moment distribution across each population of duplicate biological experiments with atleast 50 comets analyzed is depicted by a violin plot. Ordinary one-way ANOVA was performed. Adjusted *P* values: ** *P*-value < 0.001, **** *P*-value < 0.00001. (**E**) Heat map of expression (fold change) in mRNA levels of top two translesion polymerases (that were enriched in B16 microarray) (top), and a panel of known DNA replication stress response genes by qRT-PCR analysis in NHEM (Control, PTU or tyrosine treated). Data represented as mean of triplicate biological experiments. (**F**) Western blot analysis of NHEM treated with DMSO or 50 nM AZ20, a selective inhibitor of ATR kinase, for 24 h. Numbers below the blot correspond to control normalized expression of the indicated protein *wrt* beta-actin. Experiments were performed in biological duplicates. (**G**) mRNA levels of *Polk* in unpigmented B16 cells mock transfected, or with either control DNA, melanin modified DNA (plasmid DNA was incubated with L-DOPA and tyrosinase and column purified after 24 h) (Mel + DNA), DNA mixed with pre-synthesized melanin and coulmn purified [DNA+(Mel)], *in-vitro* melanin synthesis (*ex-cellulo*l-tyrosine and tyrosinase added to cell media) for 24 h or cells treated with 1 mM DHI (DHI) for 24 h. Bars represent percent mRNA levels compared to control across biological triplicates. Ordinary one-way ANOVA was performed. Adjusted *P* values: * *P*-value < 0.05. (Inset) Western blot analysis of B16 cells transfected with only DNA (Con DNA) or melanin-modified DNA (Mel + DNA) with Polκ antibody normalized to HSC70. Experiments were performed in biological triplicates.

Since B16 cells are derived from mouse melanoma, it is likely that the observed response could be restricted to melanoma cells and not a physiological response of melanocytes. Hence, we subjected already pigmented normal human epidermal melanocytes (NHEM) that are derived from healthy skin and represent primary melanocytes, to differential pigmentation with PTU and tyrosine for seven days (Figure 5A). Assessment of H2AX phosphorylation by western blot analysis revealed a similar pattern of increased phosphorylation and a concomitant increase in total protein level with hyperpigmentation (Figure 5A). This was recapitulated in the foci formation detected by immunofluorescence analysis (Figure 5B, C). Alkaline comet assay revealed that the DNA breaks were highest in hyperpigmented cells and lowest in depigmented cells (Figure 5D). Hence, these observations indicate that pigmentation is indeed a strong physiological response that causes DNA breaks in non-transformed primary cells. We therefore propose that melanogenesis is potentially genotoxic and causes DNA damage in melanocytes.

Subjecting primary melanocytes with varying levels of pigmentation to qRT-PCR analysis for *POLH* and *POLK* as well as the battery of ‘replication stress response genes’ identified these DNA repair genes to be differentially expressed (Figure 5E). While *POLK* showed a correlation with pigmentation response, *POLH* showed an opposite trend. Validating the B16 based observations, several of the replication stress response genes were elevated in primary melanocytes treated with tyrosine, strengthening the possibility of DNA damage associated with a Polκ response elicitation at the RNA level. Polκ at the protein level was induced upon hyperpigmentation and reduced upon depigmentation in cultured primary human melanocytes (Figure 5A). Involvement of ATR-CHK1 axis in this induction was confirmed using ATR inhibitor AZ20 (Figure 5F). As the control primary cells are constitutively pigmented, the reduction observed with AZ20 was lower as compared to B16 cells wherein the progressive pigmentation could be induced in presence of the inhibitor.

We then assessed the ability of melanin modified DNA to elicit a Polκ response in melanocytes that do not make melanin. Transfection of plasmid DNA incubated with melanin synthesis reaction (column purified after 16 h of incubation) in depigmented B16 cells induced Polκ protein expression (Figure 5G). We could observe the induction of *Polκ* at the RNA level in these cells only when transfected with plasmid DNA incubated with melanin synthesis reaction or extracellular melanogenesis achieved with 1mM tyrosine and tyrosinase enzyme in the culture medium. Whereas, mere incubation of pre-synthesized melanin with plasmid DNA just before transfection, pre-synthesized melanin alone, or melanin synthesis reaction mixture purified through the column did not elicit Polκ response in these depigmented cells. Similar to the RNA level changes we could confirm melanin-modified plasmid DNA to elevate protein levels of Polκ (Figure 5G, inset). The melanin intermediate DHI, that caused DNA breaks in B16 cells (Figure 1F), was also able to increase *POLK* mRNA expression. Hence, the response of melanocytes to pigmentation is the induction of Polκ through melanin-mediated DNA damage. We then proceeded to investigate the role played by Polκ in maintaining genome integrity and cellular homeostasis during sustained pigmentation.

### Silencing of polκ increases DNA damage caused by melanin

**Figure 6.**
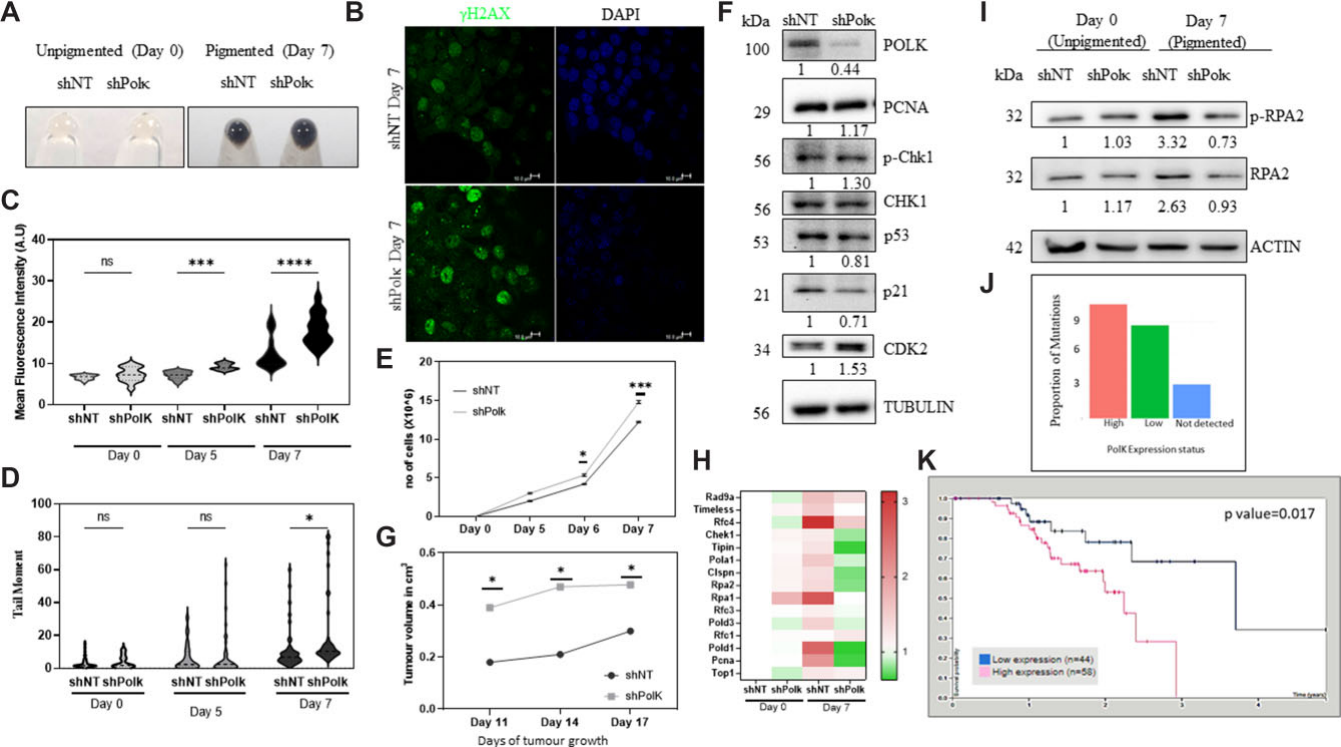
Silencing of Polκ during pigmentation prevents replication stress response despite elevated DNA damage. (**A**) Cell pellets of control non-targeting (shNT) and Polκ silenced (shPolκ) B16 cells on day 0 (left) and day 7 (right) of pigmentation. (**B**) Immunofluorescence analysis of day 7 pigmented shNT and shPolκ cells with phosphorylated H2AX antibody (puncta labelled in green) and the nucleus is counterstained with DAPI (blue). Scale bars represent 10 μm. (**C**) Quantitation of mean fluorescence intensity of γH2AX (shown in B) from two biological replicates of shNT and shPolκ cells across day 0, 5 and 7 of pigmentation induction. Two-way ANOVA was performed for multiple comparisons. Adjusted *P* values * *P*-value < 0.05 ***P-value < 0.0005 **** *P*-value < 0.00001 ns non-significant. (**D**) shNT and shPolκ expressing pigmented B16 cells were subjected to single cell electrophoresis and comet analysis (alkaline comet) on days 0, 5 and 7 of pigmentation induction. Mean tail moment distribution across each population of duplicate biological experiments with at least 50 comets analyzed is depicted by a violin plot. Two-way ANOVA was performed for multiple comparisons. Adjusted *P* values, ns non-significant, * *P*-value < 0.05. (**E**) Growth curve analysis of shNT and shPolκ expressing B16 cells on days 0, 5, 6 and 7 of pigmentation. Each point represents mean ± SEM across biological triplicates. Two-way ANOVA was performed. Adjusted *P* values: * *P*-value < 0.05, *** *P*-value < 0.001. (**F**) Western blot analysis of DNA repair and cell cycle related proteins in shNT and shPolκ cells. Numbers below represent tubulin normalized fold changes *wrt* shNT. Experiments were performed in biological duplicates. (**G**) shNT and shPolκ expressing B16 cells were injected inside the flank of C57/BL6 mice and allowed to grow as tumors. The volume of the tumor was non-invasively monitored and plotted over time of biological triplicates mean ± SEM. Two-way ANOVA was performed for multiple comparisons. Adjusted *P* values: * *P*-value < 0.05. (**H**) Heat map of expression (fold change) in mRNA levels of a panel of known DNA replication stress response genes by qRT-PCR analysis in shNT (day 0-unpigmented and day 7-pigmented) and shPolκ (day 0-unpigmented and day 7-pigmented) B16 cells. (**I**) Western blot images and analysis of p-RPA2 and total RPA2 in shNT and shPolκ B16 cells (day 0 unpigmented and day 7 pigmented). Numbers below represent beta-actin normalized fold changes *wrt* shNT at day 0. Experiments were performed in biological triplicates. (**J**) Analysis of melanoma samples from TCGA data for mRNA expression of *POLK* (high, low or not detected) segregated into bar plots and proportion of mutations were plotted on y-axis. (**K**) Survival plot of melanoma patients with low or high expression of *POLK* from TCGA data. Analysis from Human Protein Atlas database. Paired *t*-test *P* value 0.017.

We resorted to silencing Polκ in B16 cells stably expressing shRNA targeting Polκ (shPolκ) and this was compared to non-targeting shRNA against luciferase (shNT). B16 cells were freshly transfected and used as an enriched pool to prevent multiple consequences of Polκ depletion (33). Under pigmenting conditions both shNT and shPolκ showed comparable pigmentation by day 7 (Figure 6A). We observed that the depigmented day 0 cells had minimal γH2AX puncta, and these were comparable across shNT and shPolκ cells. Pigmented day 7 shPolκ cells have more mean fluorescence intensity of the foci indicating higher DNA damage (Figure 6B). With progressive pigmentation the level of DNA damage increased more in the shPolκ cells compared to shNT cells (Figure 6C). Similarly, single stranded DNA breaks and alkali sensitive (abasic) sites assessed by comet assay showed a progressive increase in the mean tail moment with pigmentation. This increase was more in the shPolκ cells on day 7 when the pigmentation was the highest (Figure 6D). Sequence independent silencing RNA (siRNA) also confirmed this increase in mean tail moment in pigmented cells (Supplementary Figure S5B). shPolκ cells resulted in an increase in the number of abasic sites, confirming a role for Polκ in resolving these lesions (Supplementary Figure S5C). Abasic sites arise from melanin-modifications (Figure 1C), and this result confirms a critical role for Polκ in its repair perhaps by its TLS activity. These results indicate that Polκ is required to resolve the DNA damage caused by melanin.

### Polκ is necessary to mount a replication stress response in pigmented melanocytes

So far, we were able to establish that melanin causes DNA damage that consequently delays the progression into cell cycle (Figure 1C, top & Supplementary Figure S2B). Pigmenting melanocytes elicit induction of Polκ as a response through ATR-CHK1 axis (Figure 3E). Further, silencing studies indicated that Polκ is necessary to keep a check on the DNA damage caused during pigmentation. Based on these results we anticipated that silencing of Polκ would result in more cell cycle arrest and cell death as a consequence.

Interestingly, the number of cells were significantly higher upon Polκ knockdown during progressive pigmentation (Figure 6E). While the growth differences were non-significant at earlier days, the differences become prominent on day 7, when the pigmentation was highest. Cell cycle analysis revealed an increase in S + G2/M population suggesting aberrant progression into cell cycle despite pigmentation-induced DNA damage (Supplementary Figure S5D). Additionally, we verified that pigmented shNT and shPolκ cells exhibited comparable PI-stained populations, indicating similar cell viability, and the annexin-V labeled population showed similar levels of apoptosis between them (Supplementary Figure S5E, F). This unexpected observation prompted us to look at the battery of proteins associated with cell cycle. A panel of orchestrators and effectors were analyzed by western blot on day 7 of pigmentation upon knockdown of Polκ. Confirming the silencing, levels of Polκ protein was downregulated (Figure 6F). PCNA is an effector of Polκ and a marker of proliferating cells. However, upon Polκ silencing PCNA levels remained unaltered, perhaps due to opposing effects of proliferation and the absence of key repair polymerase. However, both p53 and p21 that function to couple DNA damage to cell cycle arrest were low and CDK2 was elevated, further substantiating that more cells are in the proliferative phase of cell cycle. This aberrant proliferation was further validated with injection of shNT and shPolκ cells into the flank of C57/BL6 mice to grow autologous pigmented tumors. We consistently observed that shPolκ cells formed tumors with higher volume, confirming that under this pigmenting condition as well, insufficient induction of Polκ results in lack of cell cycle arrest (Figure 6G, Supplementary Figure S5G, H). Hence, Polκ knockdown does not result in cell cycle arrest or cell death as anticipated, instead the DNA-damaged cells continue to proliferate more.

We then assessed the replication stress response genes and observed a pronounced induction in pigmented day 7 shNT cells as observed earlier for control B16 cells (Figures 6H, 2B). Pigmented day 7 shPolκ cells failed to mount such a response and several of the genes were mildly downregulated compared to shNT day 0 cells. Indicating that Polκ is a crucial elicitor of replication stress response when cells are challenged with melanin modified DNA. This was further strengthened by the loss of induction of phosphorylated and total RPA2 levels in day 7 shPolκ cells (Figure 6I).

In the absence of Polκ, despite the presence of elevated DNA damage, cells aberrantly progress through the cell cycle and proliferate more. This could potentially render these pigmenting cells vulnerable to genome instability. Substantiating this possibility, a recent systematic investigation of combination of DNA damaging agents and repair gene knockouts carried out in *C. elegans* highlighted an increased mutation frequency for alkylating agents MMS and DMS upon deletion of the Polκ ortholog (34). In the context of melanocytes, we would expect that Polκ would be the primary mitigating response to melanin-induced DNA damage, instead we observe that Polκ is the primary mediator of cellular response caused by this damaged DNA. At a molecular level the mechanism by which a translesion polymerase like Polκ would be able to elicit a replication stress response remains enigmatic. It is likely that Polκ based flagging of melanin lesions may elicit this response by invoking other repair proteins like CHK1.

### Signatures of somatic mutations in human melanoma with varying POLK levels

Having established the role of sustained melanogenesis in causing DNA damage and mapping the Polκ response by melanocytes, we decided to explore whether this is relevant in the human melanoma context. We extracted the cutaneous melanoma samples from The Cancer Genome Atlas (TCGA) and segregated the samples based on the mRNA expression of *POLK* (35). While 83 melanoma samples did not have detectable expression of *POLK* mRNA by sequencing, FPKM based binning resulted in 207 patients with high levels of *POLK* and 175 patients had lower expression of *POLK* (36). Overall number of somatic mutations were highest in high-*POLK* group and least in samples that did not have detectable *POLK* (Figure 6J). A systematic two group comparison of somatic mutation burden using Welch Two Sample t-test in *POLK* expressing (high + low) with *POLK* non-detected group resulted in a *P*-value < 0.08. The mean in group ‘expressed’ is 505 whereas the mean in group ‘not-expressed’ is 380. Thereby, suggesting that expression of *POLK* could explain a fraction of mutations in human melanoma, and provide physiological relevance to the observations in cultured cells. The survival plot of patients for whom the *POLK* mRNA data was available (58 high and 44 low) suggested a trend with higher mortality in the high *POLK* group compared to low *POLK* group (*P*-value < 0.05), which could be explained by the error prone nature of Polκ (Figure 6K). The status of pigmentation of melanoma (compared to cognate skin-derived melanocytes) was not available in the TCGA meta-data, to empirically assess this we looked at the expression of several pigmentation genes for which the data was available. We observed that most of the pigmentation genes were not significantly different across the three groups, suggesting similar distribution of pigmentation (Supplementary Figure S5G).

Subsequently, our focus shifted towards identifying distinct signatures within the array of somatic mutational changes associated with varying levels of Polκ expression. In a prior study, we had already compared the somatic mutational spectrum of different cutaneous malignancies with both lesional and non-lesional vitiligo skin samples (37). In this investigation, we reanalyzed the data, specifically focusing on melanoma samples from TCGA, grouped according to their respective Polκ expression levels. Notably, SBS7a and SBS7b were prevalent in both groups of melanoma samples, as well as in various other cutaneous tissues, including healthy skin (Supplementary Figure S5J). These signatures were likely linked to sun exposure, a well-known factor in cutaneous mutational patterns (20,38,39).

Moreover, we identified additional signatures enriched in melanoma samples. Among them, SBS6 and SBS21 were enriched exclusively in low Polκ tumors, and interestingly, they are associated with genome instability. Conversely, SBS31 was found to be enriched solely in high Polκ tumors, and it is linked to prior chemotherapy with Platinum drugs, which induce DNA adducts, resembling the proposed mechanism involving melanin in this study. Although it would have been intriguing to explore the correlation of SBS signatures with Polκ levels in the context of patients' prior chemotherapy history, unfortunately, due to a lack of available information, we could not establish conclusive evidence in this regard.

Thereby, in this study we reinforce the correlation of DNA damage and possible mutations with pigmentation. This provides a compelling reason to further investigate this aspect of eumelanogenesis and its implication in melanoma in future.

## DISCUSSION

While the UV protection role is likely to be overarching, the current work unravels a rather unwanted side-effect during synthesis of the esoteric biopolymer melanin. This was earlier alluded to, but the details of the DNA damage and the response elicited by melanocytes remained elusive so far (5,6). In this work, using cell-bases models, we demonstrate that uncontrolled melanin synthesis, *via* its intermediates causes DNA damage. The key response of pigmenting cells is the replication stress response which critically involves induction of translesion polymerase Polκ *via* the ATR-Chk1 signaling axis.

Among translesion repair enzymes, Polκ is necessary to bypass large bulky adducts on the nucleotide bases that are not amenable for repair by other DNA repair systems (40,41). Additionally other non-canonical, non-TLS functions of Polκ such as stabilizing replication forks *via* Chk-1 and repair of oxidized dNTPs are well-known (42). Herein we report yet another unexpected, crucial cellular function of Polκ in eliciting replication stress response in melanocytes undergoing pigmentation induced DNA damage. Supported by the co-localization of EdU and γH2AX positive foci in pigmented cells, our initial notion was that the role of Polκ would be restricted to translesional bypass of melanin modified DNA adducts. Yet, only a minor proportion of cells (approximately 15%) exhibited these double positive foci, suggesting that Polκ may have an additional role in the majority of pigmenting cells. Some TLS enzymes such as Polζ help cancer cells come out of replication stress (39). However, Polκ elicits replication stress response upon melanin-induced DNA damage. This ‘checkpoint controller’ role of Polκ would permit enough time for melanocytes to repair melanin-damaged DNA by TLS and possibly other repair systems before the next round of cell cycle is initiated. This is strikingly different to the reported role of TLS enzymes as a mitigator of replication stress (39). This could be attributed to the melanin-based DNA lesions, as opposed to hydroxyurea induced replication fork stalling, as the cause of replication stress (40). It is also likely that additional factors associated with melanogenesis such as protein oxidation could also contribute to the replication stress response and this would need to be independently assessed (43).

In an earlier study, authors report subcellular localization of Polκ inside nucleus of human melanoma cells and this involves mTOR pathway (44). The work further demonstrated Polκ to provide survival advantage upon pharmacological inhibition of the oncogenic BRAF, independent of creating somatic mutations in the genome. In their study, overexpression of Polκ did not significantly increase the mutation burden. However, this remains to be seen in the context of ongoing melanogenesis. We do observe that in our study the induction of Polκ in response to melanogenesis is independent of mTOR pathway, as the pharmacological inhibition by PP242 does not alter Polκ levels (Supplementary Figure S3E). In another study, role of Polκ in promoting DNA synthesis and recovery of replication stress at stalled forks upon nucleotide deprivation was elucidated (40). The link between CHK-1 and Polκ seems to be 2-fold, while in some cells loss of Polκ abrogates CHK-1 phosphorylation (42,45), whereas in multiple cell types knock down of Polκ did not alter CHK-1 phosphorylation (46). In these studies, effect of CHK-1 phosphorylation on Polκ induction is not systematically investigated. Our observations highlight a third interplay wherein Polκ induction is under the control of ATR-CHK-1 axis. Functions of Polκ during DNA damage response may be different to its homeostatic role in DNA fork stalling. Cells seem to have multiple regulatory check points at the level of *Polκ* gene expression as well as its nuclear localization, possibly due to its error-prone nature and/or its crucial role in replication stress response activation.

Most of the work on UV-induced melanoma formation has concentrated on direct DNA damage. This is substantiated by the prevalence of UV-signature mutations in several melanoma samples. This link is further strengthened by the recent data suggesting an increase in the incidence of melanoma among users of artificial tanning beds (47). Surprisingly, despite deep-seated within the epidermis and loaded with melanin, melanocytes are not that well-protected from UV and are vulnerable to DNA damage. It is apparent that there are both UV-signature mutations and unexplained large-scale genomic alterations associated with malignant cells that could be as important as point mutations. Specifically, the known key oncogenic mutations in melanoma including, BRAF V600E and NRAS Q61L/R, do not have the UV-signature (48). V600E mutation involves T > A transversion and could be ascribed to the propensity of Polκ to insert A against a lesion. Suggesting that melanin damage followed by translesion bypass by Polκ could account for at least a fraction of the known mutations in melanoma. It is likely that UV-induced melanogenesis could result in these driver mutations adding a layer of complexity to the link between UV and melanoma. While the activation of melanocytes in conditions such as tanning enable adaptive pigmentation response, repeated or uncontrolled activations could alter these cells and possibly promote mutagenesis and facilitate their transformation into melanoma. The role of Polκ in restoring melanocyte homeostasis seems to be 2-fold, with TLS and more importantly as a check point controller. While the former may involve some errors, the latter effect of Polκ is primarily protective against deleterious mutations, providing reasons for its evolutionary conservation. Thereby, our study establishes a hitherto unknown, undesirable side of melanin and its possible involvement in melanoma.

## Supplementary Material

gkad704_Supplemental_files

## Data Availability

The data underlying this article are available in the article and in its online supplementary material.
