# Supplementary material for: Sustained pigmentation causes DNA damage and invokes translesion polymerase Polκ for repair in melanocytes
Source: Nucleic Acids Res. 2023 Sep 11;51(19):10451–66. doi: 10.1093/nar/gkad704 (PMC10602914; doi:10.1093/nar/gkad704)
Supplement: gkad704_Supplemental_files [file gkad704_supplemental_files.zip › Supplementary Figures.pdf]

Supplementary Figures

Sustained pigmentation causes DNA damage and invokes translesion polymerase Polκ for repair in melanocytes

Madeeha Ghazi[1,2†], Shivangi Khanna[1,2†], Yogaspoorthi Subramanian[1,2], Jeyashri Rengaraju[1,2], Farina Sultan[1,2], Iti Gupta[1,2], Kanupriya Sharma[3], Sudhir Chandna[3], Rajesh S Gokhale[1,4] and Vivek T Natarajan[1,2*]

[1] CSIR-Institute of Genomics and Integrative Biology, Mathura Road, New Delhi, India

[2] Academy of Scientific and Innovative Research (AcSIR), Ghaziabad, Uttar Pradesh, India

[3] Institute of Nuclear Medicine and Allied Sciences, Defence Research and Development Organization, Delhi, India

[4] National Institute of Immunology, Aruna Asaf Ali Marg, New Delhi, India

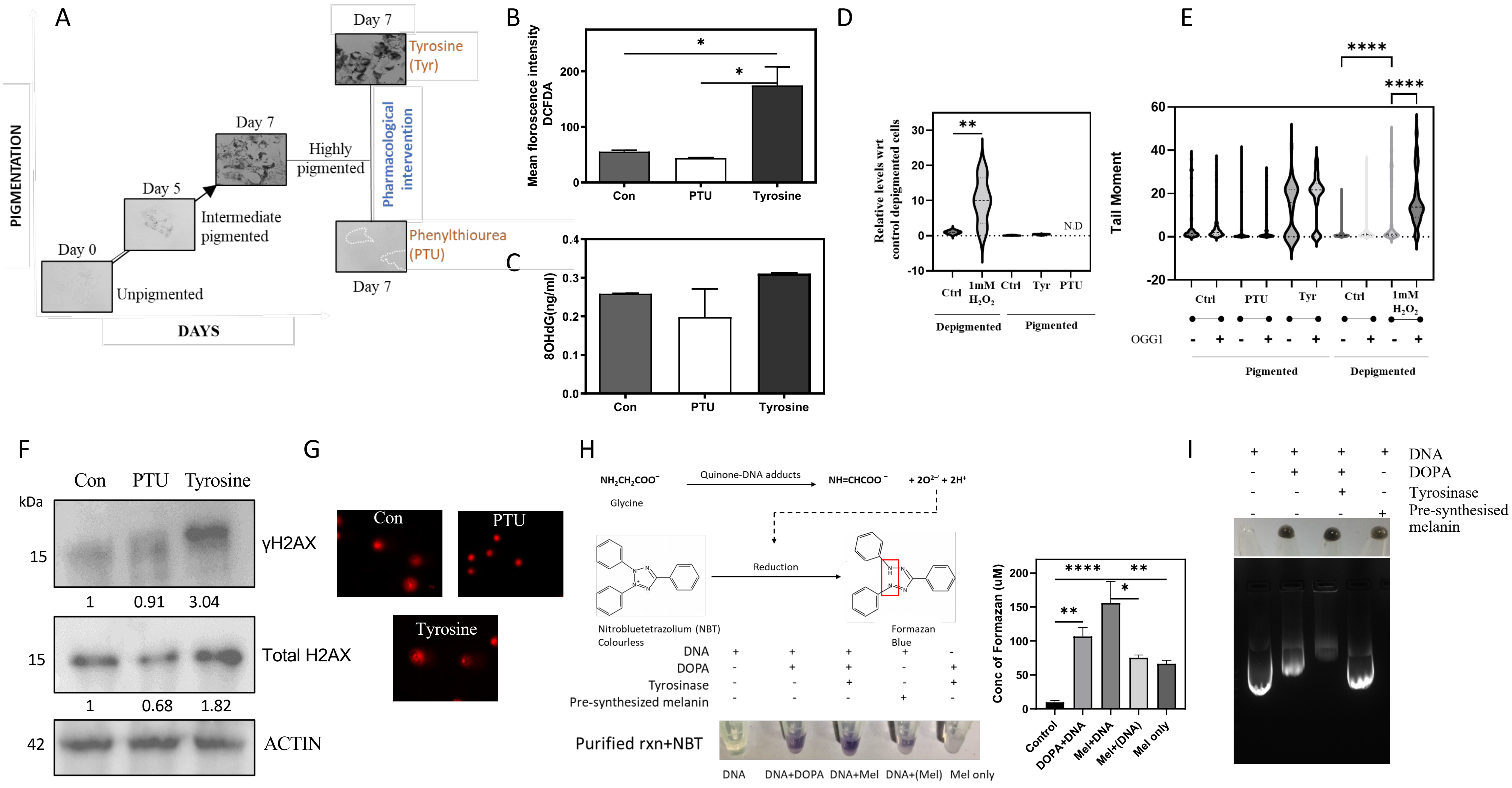
A
PIGMENTATION
DAYS
Day 0
Unpigmented
Day 5
Intermediate pigmented
Day 7
Highly pigmented
Pharmacological intervention
Day 7
Tyrosine (Tyr)
Phenylthiourea (PTU)
B
Mean floroscence intensity DCFDA
Con
PTU
Tyrosine
C
8OHdG(ng/ml)
D
Relative levels wrt control depigmented cells
N.D
Depigmented
Pigmented
E
Tail Moment
OGG1
Pigmented
Depigmented
F
Con PTU Tyrosine
kDa
γH2AX
1 0.91 3.04
Total H2AX
1 0.68 1.82
ACTIN
G
Con
PTU
Tyrosine
H
Quinone-DNA adducts
Glycine
Reduction
Nitrobluetetrazolium (NBT) Colourless
Formazan Blue
DNA
DOPA
Tyrosinase
Pre-synthesized melanin
Purified rxn+NBT
DNA DNA+DOPA DNA+Mel DNA+(Mel) Mel only
Conc of Formazan (uM)
Control
DOPA+DNA
Mel+DNA
Mel+(DNA)
Mel only
I
DNA
DOPA
Tyrosinase
Pre-synthesised melanin

Figure S1

Supplementary Figure S1:

Effect of melanogenesis on DNA damage

A. Schematic of the experimental design of B16 progressive pigmentation model. Day 0 represents depigmented, day 5 intermediate pigmented and day 7 highly pigmented cells. Pigmentation was further altered in these cells by the use of tyrosinase inhibitor Phenylthiourea (PTU) or its substrate L-tyrosine (Tyr). (Right) These treatments given at day 1 which further enhances or decreases pigmentation across days in B16 progressive model of pigmentation.
B. Cellular ROS content was assayed with 2',7'–dichlorofluorescin diacetate (DCFDA) staining and mean fluorescence value across duplicate biological experiments is depicted as a bar plot. Bars represent mean ± SEM. Ordinary one-way ANOVA was performed Adjusted p values * p val< 0.05.
C. ELISA based 8-OHdG estimation in control, PTU and tyrosine treated cells on day 7 of pigmentation induction across two biological experiments.
D. Relative estimation of intact DNA by quantitative PCR across four loci (Mitf, Tyr, Dct and RnaseK), with and without treatment with OGG-1 (8-oxoguanine DNA glycosylase-1) represented as a bean plot. Tukey's multiple comparison test resulted in a p value <0.01 for H_2O_2 treatment and all other comparisons were not significant.
E. OGG-1 modified comet assay was performed on differentially pigmented B16 cells and for comparison, unpigmented B16 cells were treated with H_2O_2. Statistical analysis was performed by Tukey's multiple comparison test that resulted in significant p value only for H_2O_2 treatment.
F. Western blot analysis of control, PTU or tyrosine treated day 7 B16 cells with γH2AX antibody, total H2AX and beta actin. Experiments were performed in biological duplicates.
G. PTU and tyrosine treated day 7 pigmented B16 cells were subjected to single cell electrophoresis and comet analysis, representative images of PI-stained comets are depicted.
H. NBT (Nitro Blue Tetrazolium) assay: Formazan based detection of quinone-DNA adducts by reduction of NBT. (top) Mechanism of NBT assay is depicted (bottom) Sample images of differentially treated plasmid DNA (details in materials and methods) after incubation with NBT, with blue color formation indicating the formation of formazan by quionone-DNA adducts. (right) Absorbance based estimation of the concentration of formazan (µM) present in each sample. Bars represent mean ± SEM across two biological replicates. Ordinary one-way ANOVA was performed. Adjusted p values * p val< 0.05 **p val<0.001 **** p val<0.0001.
I. (top) Images of samples after incubation of plasmid DNA (without purification through column) as indicated. (bottom) Agarose gel electrophoretic mobility assessment upon treatment of plasmid DNA with either L-DOPA, L-DOPA with tyrosinase (*in-vitro* melanin synthesis reaction) or with pre-synthesized melanin.

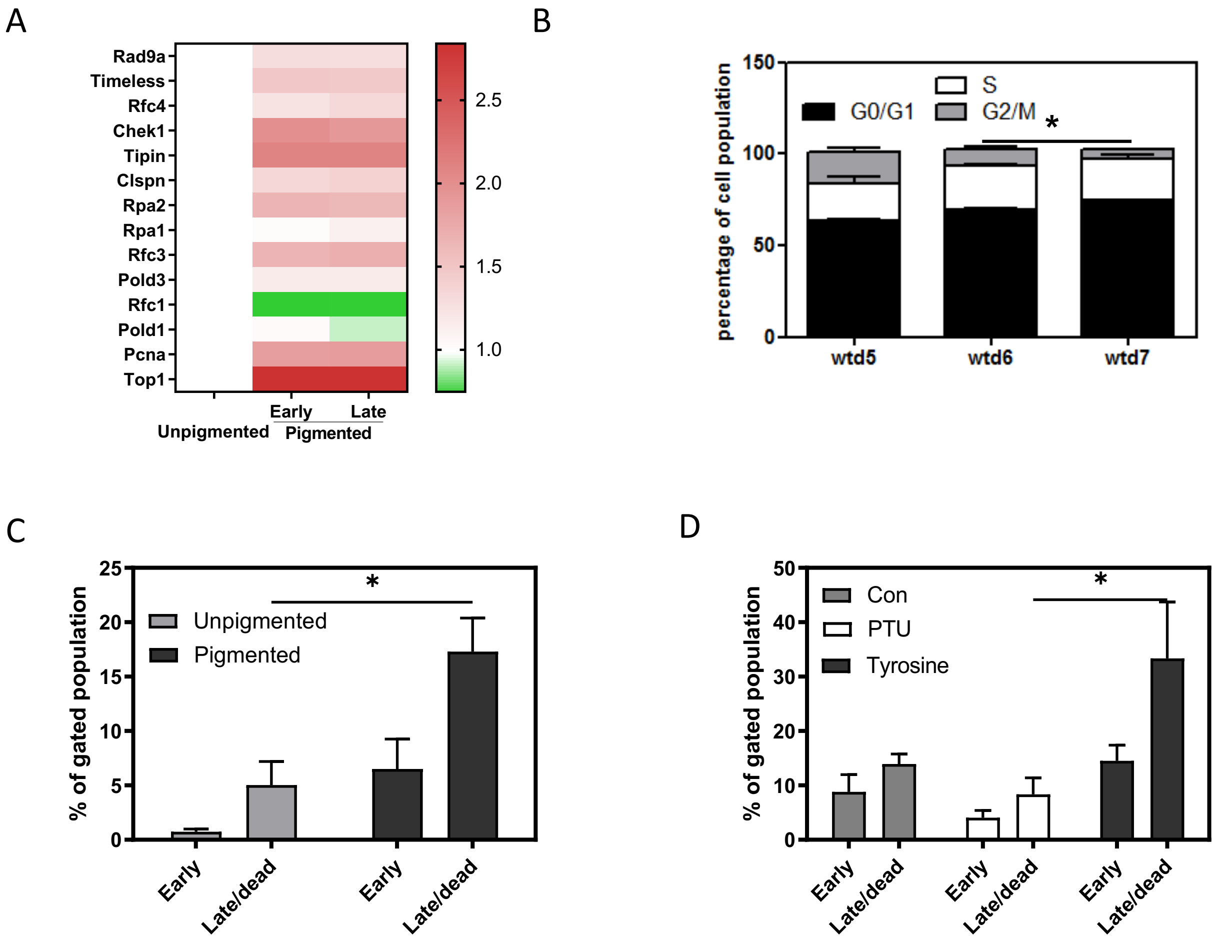
A
Rad9a
Timeless
Rfc4
Chek1
Tipin
Clspn
Rpa2
Rpa1
Rfc3
Pold3
Rfc1
Pold1
Pcna
Top1
Early
Late
Unpigmented
Pigmented
2.5
2.0
1.5
1.0
B
percentage of cell population
150
100
50
0
G0/G1
S
G2/M
*
wtd5
wtd6
wtd7
C
% of gated population
25
20
15
10
5
0
Unpigmented
Pigmented
*
Early
Late/dead
Early
Late/dead
D
% of gated population
50
40
30
20
10
0
Con
PTU
Tyrosine
*
Early
Late/dead
Early
Late/dead
Early
Late/dead

Figure S2

Supplementary Figure S2:

Uncontrolled melanogenesis causes replication stress response and apoptosis

A. Gene expression by B16 cells pigmentation microarray analysis of a panel of known DNA replication stress genes at day 0 (unpigmented), early and late pigmented cells. The heat map represents fold change wrt unpigmented cells).
B. Cell cycle analysis of B16 cells at day 5, day 6 and 7 of pigmentation. Stacked bars represent mean ± SEM across two biological replicates. Two-way ANOVA was performed. Adjusted p value * p val< 0.05.
C. Estimation of early apoptotic (annexin V positive population) and late apoptotic/dead cells (annexin V and PI double positive population) in unpigmented (day 0) and pigmented (day 7) B16 cells. Bars represent mean ± SEM across two biological replicates. Ordinary one-way ANOVA was performed. Adjusted p values * p val< 0.05.
D. Estimation of early apoptotic (annexin V positive population) and late apoptotic/dead cells (annexin V and PI double positive population) upon differential pigmentation (PTU and tyrosine). Bars represent mean ± SEM across two biological replicates. Ordinary one-way ANOVA was performed. Adjusted p values * p val< 0.05.

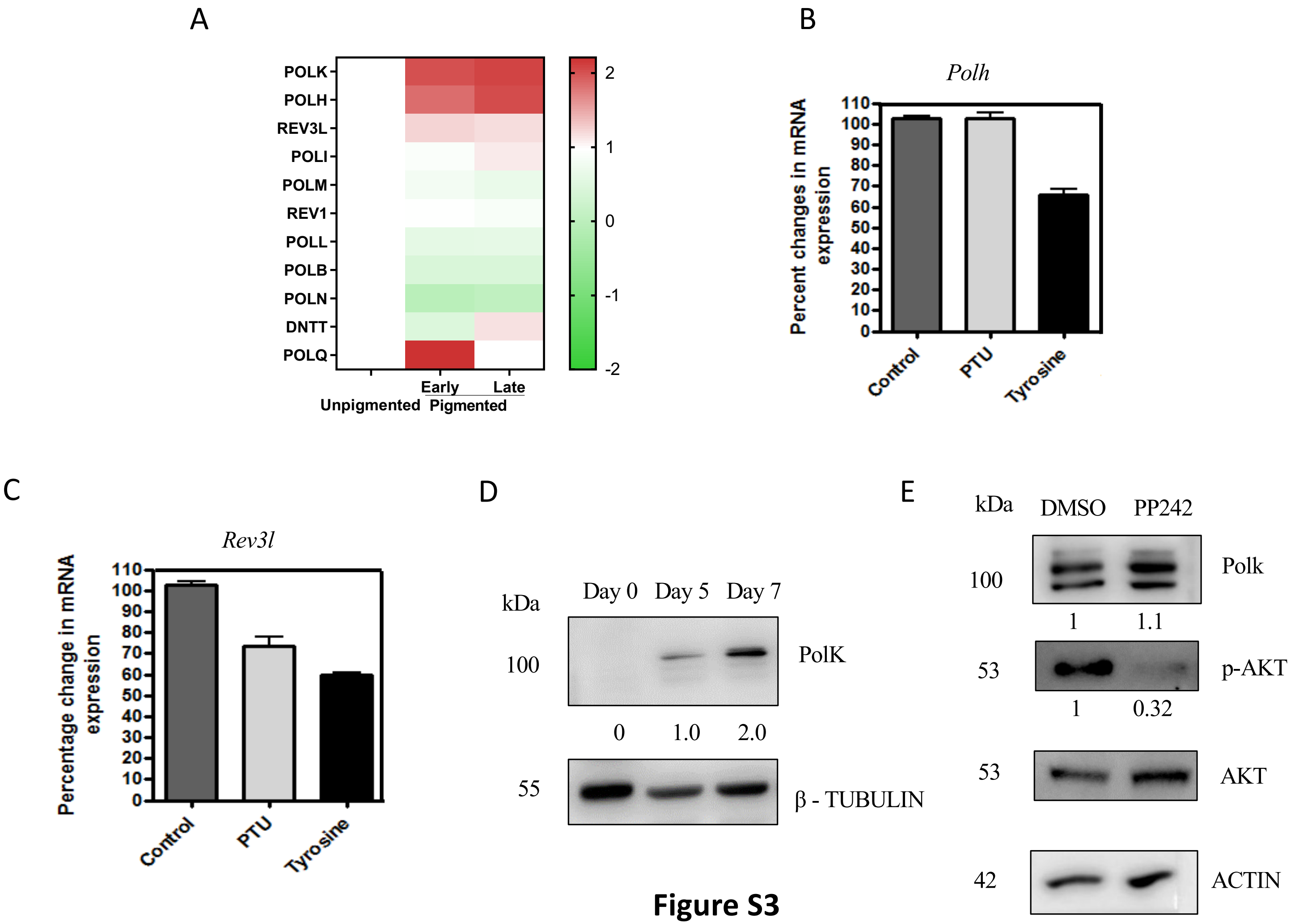
A
POLK
POLH
REV3L
POLI
POLM
REV1
POLL
POLB
POLN
DNTT
POLQ
2
1
0
-1
-2
Early
Late
Unpigmented
Pigmented
B
Polh
Percent changes in mRNA expression
110
100
90
80
70
60
50
40
30
20
10
0
Control
PTU
Tyrosine
C
Rev3l
Percentage change in mRNA expression
110
100
90
80
70
60
50
40
30
20
10
0
Control
PTU
Tyrosine
D
kDa
Day 0
Day 5
Day 7
100
PolK
0
1.0
2.0
55
β - TUBULIN
E
kDa
DMSO
PP242
100
Polk
1
1.1
53
p-AKT
1
0.32
53
AKT
42
ACTIN

Figure S3

Supplementary Figure S3:

Polκ is elicited as a key DNA translesion polymerase upon melanogenesis induction

A. Heat map of expression changes of all translesion polymerases during different days of pigmentation in B16 cells based on our earlier published microarray data (GSE54359)
B. Real-time qRT-PCR analysis of *Polh* translesion polymerase in differentially pigmented B16 cells treated with PTU and Tyr. Bars represent mean ± SEM across two biological replicates.
C. Real-time qRT-PCR analysis of *Rev3l* translesion polymerase in differentially pigmented B16 cells treated with PTU and Tyr. Bars represent mean ± SEM across two biological replicates.
D. Western blot analysis of B16 cells at different days of pigmentation with Polκ and beta Tubulin antibodies. Numbers represent relative fold change *wrt* beta Tubulin. Experiment was performed in biological duplicates.
E. Western blot analysis of pigmented B16 cells with Polκ, p-AKT and total AKT normalized with beta. Cells were treated with mTOR inhibitor PP242. Numbers represent relative fold change *wrt* beta actin. Experiment was performed in biological triplicates.

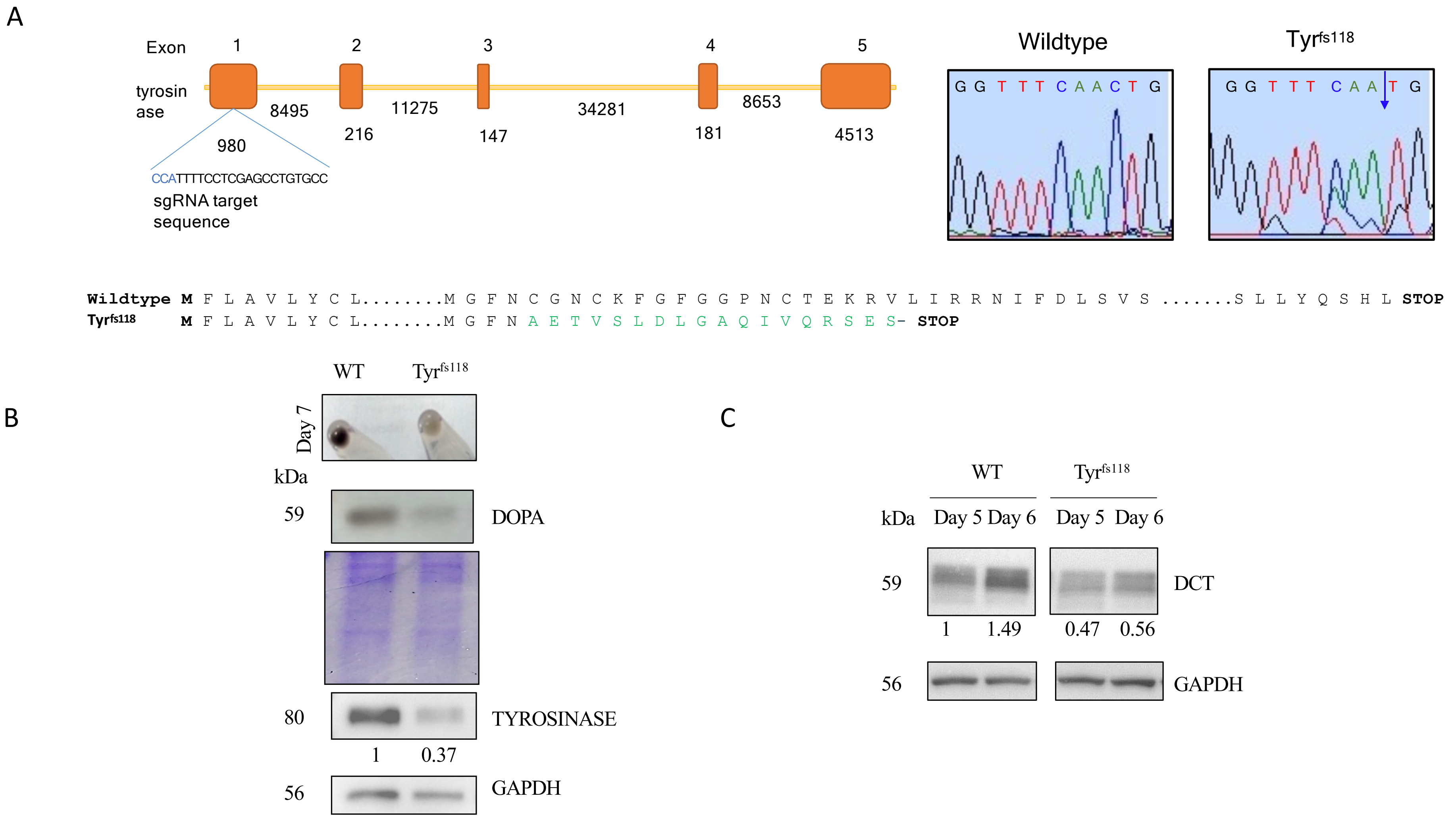
A
Exon
1
2
3
4
5
tyrosinase
8495
11275
34281
8653
980
216
147
181
4513
CCATTTTCCTCGAGCCTGTGCC
sgRNA target sequence
Wildtype
GGTTTCAACTG
Tyrfs118
GGTTTCAATG
Wildtype M F L A V L Y C L........M G F N C G N C K F G F G G P N C T E K R V L I R R N I F D L S V S ....S L L Y Q S H L STOP
Tyrfs118 M F L A V L Y C L........M G F N A E T V S L D L G A Q I V Q R S E S- STOP
B
WT
Tyrfs118
Day 7
kDa
59
DOPA
80
TYROSINASE
1
0.37
56
GAPDH
C
WT
Tyrfs118
kDa
Day 5
Day 6
Day 5
Day 6
59
DCT
1
1.49
0.47
0.56
56
GAPDH

Figure S4

Supplementary Figure S4:

Targeted ablation of Tyrosinase locus with CRISPR to generate a genetic model of hypopigmentation

A. (Top left): The mouse Tyrosinase (Tyr) gene architecture and the position of single guide RNA (sgRNA) sequence. (Top right): Sanger sequencing chromatogram of wild type (B16 WT) and tyrosinase mutant clone (B16 Tyr^{fs118}). Deletion of C nucleotide at position 459 is indicated by the blue arrow. (Bottom): Predicted amino acid sequence of WT and Tyr^{fs118} coding region. In the mutant sequence, a premature stop codon is made which would result in the formation of truncated protein.

B. (Top): Cell pellet images of day 7 B16 WT and Tyr^{fs118} cells. (Middle): Representative image of L-DOPA zymography along with its representative Coomassie stained gel image. (Bottom): Western blot image of tyrosinase protein in day 7 B16 WT and Tyr^{fs118} cells normalized with GAPDH. Experiments were performed in biological duplicates.

C. Western blot image of DCT in day 6 and 7 B16 WT and Tyr^{fs118} cells. Numbers represent relative fold change *wrt* GAPDH. Experiments were performed in biological duplicates.

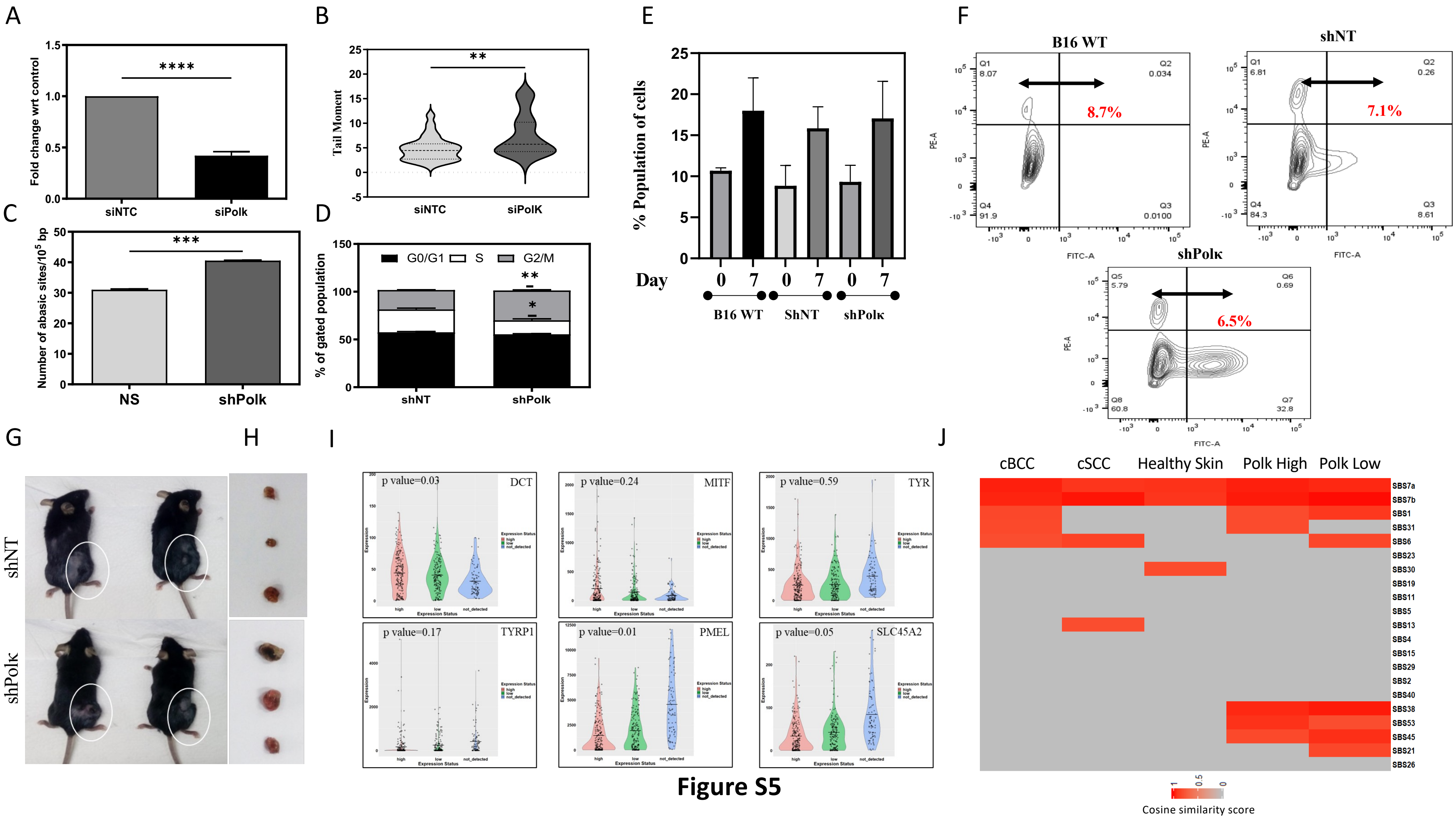

A
Fold change wrt control
****
siNTC
siPolk
B
Tail Moment
**
siNTC
siPolK
C
Number of abasic sites/10^5 bp
***
NS
shPolk
D
% of gated population
G0/G1 S G2/M
**
*
shNT
shPolk
E
% Population of cells
Day
0 7 0 7 0 7
B16 WT
ShNT
shPolκ
F
B16 WT
8.7%
shNT
7.1%
shPolκ
6.5%
PE-A
FITC-A
G
shNT
shPolκ
H
I
p value=0.03
DCT
p value=0.24
MITF
p value=0.59
TYR
p value=0.17
TYRP1
p value=0.01
PMEL
p value=0.05
SLC45A2
J
cBCC
cSCC
Healthy Skin
Polk High
Polk Low
SBS7a
SBS7b
SBS1
SBS31
SBS6
SBS23
SBS30
SBS19
SBS11
SBS5
SBS13
SBS4
SBS15
SBS29
SBS2
SBS40
SBS38
SBS53
SBS45
SBS21
SBS26
Cosine similarity score

Figure S5

Supplementary Figure S5:

Silencing of Polκ increases DNA breaks and abasic sites and increases tumour volume

A. Polκ was silenced using an siRNA pool (4 different siRNAs, sequence independent of the shRNA used in this study) and real-time PCR analysis of the *Polκ* mRNA levels were measured by qRT-PCR analysis. Control cells are labelled as siNTC (transfected with a non-targeting siRNA) and knockdown cells as siPolk. Student's t-test (unpaired) was performed. **** p val < 0.00001.
B. B16 cells transfected with siNTC or siPolk siRNA pool on day 5 of pigmentation and on day 7 subjected to single cell electrophoresis and comet analysis (alkaline comet). Mean tail moment distribution across each population of duplicate biological experiments with at least 50 comets analyzed is depicted by a violin plot. Student's t-test (unpaired), ns non-significant, ** p val < 0.005.
C. Number of abasic sites in the genomic DNA of B16 cells expressing non-targeting shRNA (shNT) or Polκ silencing shRNA (shPolκ). Bars represent mean ± SEM across duplicate biological experiments. Student's t-test (unpaired)**** p val < 0.00001.
D. Cell cycle analysis by propidium iodide staining. The stack bars represent mean ± SEM across duplicate experiments. Two-way ANOVA was performed. Adjusted p values * p val< 0.05 ** p val< 0.01.
E. Detection of late apoptotic cells using Propidium Iodide staining in B16 WT, shNT (non-targeting shRNA) and shPolK in both depigmented (Day 0) and Pigmented (Day 7) conditions. Bars represent Mean ± S.D between biological triplicates.
F. Detection of early apoptotic cells using Cy3 labelled Annexin-V staining in B16, shNT (non-targeting shRNA) and shPolK pigmented conditions (Day 7).
G. Mice images on day 17 post-injection of shNT or shPolκ expressing B16 cells into the flank of C57/BL6 mice and allowed to grow as tumours. White ovals indicate the position of tumours.
H. Images of excised tumours of shNT or shPolκ silenced B16 cells injected in C57/BL6 mice.
I. Expression of DCT, MITF, TYR, TYRP1, PMEL and SLC45A2 genes in melanoma patients having high, low or not expressed *Polk* gene expression (shown in Fig 6 J).
J. Heatmap of the cosine values for the extracted signatures across multiple skin cancers including healthy skin. Legend indicates the Cosine similarity score.
